# Knowledge translation for delirium superimposed on dementia care: a realist review

**DOI:** 10.1093/geront/gnag071

**Published:** 2026-04-29

**Authors:** Yvette I-Pei Tsai, Minah Amor Gaviola, Mathieu Figeys

**Affiliations:** School of Nursing & Midwifery, University of Newcastle, Callaghan, NSW, Australia; School of Nursing & Midwifery, University of Newcastle, Callaghan, NSW, Australia; School of Nursing & Midwifery, University of Newcastle, Callaghan, NSW, Australia

**Keywords:** Delirium, Dementia, Care Practice, Knowledge Translation, Realist Synthesis

## Abstract

**Background and Objectives:**

People living with dementia are at higher risk of developing delirium during healthcare admission. While there have been advancements in delirium interventions, challenges persist in translating evidence-based knowledge into practice. This review aims to explore the contextual factors and underlying mechanisms of knowledge translation for preventing and managing delirium in people with dementia, and to understand how these processes contribute to intended outcomes.

**Research Design and Methods:**

A realist synthesis was adopted to construct context-mechanism-outcome configurations exploring the processes of knowledge translation. Five databases were searched from inception till May 2024 and updated in September 2025. Study screening and selection were performed independently, and quality was appraised based on content relevance, richness, and rigor. Data were extracted and synthesized to define and redefine the program theory using the Knowledge-to-Action cycle framework.

**Results:**

Of 4,142 articles identified, 31 studies were included. Four themes emerged: time constraints, awareness and knowledge of delirium and dementia, treatment effectiveness, and feasibility and sustainability of implementation. Eight context–mechanism–outcome configurations were constructed under the four themes: integration into existing practice, dedicated personnel, partnership with direct stakeholders, iterative education and training, effective leadership, acknowledging pharmacological and non-pharmacological mixed effects, ongoing attention to feedback and collaborative efforts.

**Discussion and Implications:**

Based on the constructed context–mechanism–outcome, recommendations for future implementation of delirium interventions were summarized to support knowledge translation in caring for older people with dementia, with or at risk of delirium. Further exploration of synthesized findings in real-world settings is needed to strengthen the evidence-informed theory.

## Background

Delirium is a common complication of healthcare admission. As an acute neuropsychiatric syndrome, delirium is characterized by the rapid onset of cognitive impairment that changes in attention, orientation, memory, abstract thinking, executive functions and behaviour (Inouye et al., 2014). Over 50% of people aged over 65 experience delirium during hospital stays, with up to 31% of cases occurring in individuals with dementia (Al Farsi et al., 2023; Nitchingham & Caplan, 2021), and 22% occurring in community and long-term care settings (de Lange et al., 2013). The causes of delirium are complex and multifactorial, involving predisposing factors (e.g., age, comorbidities, cognitive impairment/dementia) and precipitating factors (e.g., trauma, surgery, medications, environment) (Aldecoa et al., 2017). People living with dementia have a twofold risk of acquiring delirium, known as delirium superimposed on dementia (DSD) (Hölttä et al., 2014). Both delirium and dementia symptoms complicate clinical care, significantly increasing the risks of mortality, transition to institutionalization, and causing emotional and physical distress for individuals, their families, and care providers (Reynish et al., 2017; Rose et al., 2021).

Recent progress in delirium prevention and management emphasizes multidomain interventions to address predisposing and precipitating factors in at-risk older individuals (Ludolph et al., 2020). Despite recent progress, a lack of standardized diagnostic methods for recognizing delirium and a lack of consensus on pharmacological treatments for managing it persist across healthcare settings (Wilson et al., 2020). While multidomain interventions for delirium are beneficial, challenges remain in clinical practice when implementing them to care for older people with dementia, with or at risk of delirium (Nitchingham & Caplan, 2021).

Barriers to overcoming the implementation challenges are attributed to multiple levels within individual and organizational contexts. Individual levels commonly relate to knowledge, awareness, and attitude about delirium and dementia, whereas organizational levels relate to workforce development, interprofessional culture and policy constraints (Bianchi et al., 2024; Teodorczuk et al., 2012). Pressure to simultaneously care for acutely ill and emergency patients, along with other competing demands, further places a time constraint on caring for people with delirium and dementia (Eagles et al., 2022; Kennedy et al., 2020; Teodorczuk et al., 2012). While care standards and policies for delirium prevention and management are available in most healthcare settings (Teodorczuk et al., 2023). It is estimated that about 60% of the care provided is thought to align with the best practice, 30% is wasteful, and 10% is considered harmful (Braithwaite et al., 2020).

Knowledge translation (KT) is conceptualized by a dynamic and iterative process of synthesis, dissemination, and exchange of ethically sound application of knowledge to improve health, services/products and strengthen healthcare systems (Straus et al., 2009). The core elements of KT involve the use of *knowledge creation* (e.g., primary research), *knowledge distillation* (e.g., creation of reviews and guidelines), and *knowledge dissemination* (e.g., publication and presentation) to inform health policy, practice, and decision-making for the improvement of healthcare outcomes (Flynn et al., 2023; Straus et al., 2009). However, translating evidence-based knowledge into real-world practices is a complex process confounded with dynamic components that interact and operate at different levels within social, cultural and economic contexts (Prihodova et al., 2019). The current advancements in multidomain interventions and persistent challenges of delirium in older people with dementia indicate that the actual use of knowledge in practice will need to move beyond the barriers and facilitators identified in the literature.

This review aims to explore the contextual factors and underlying mechanisms of knowledge translation for preventing and managing delirium in older people with dementia, and understand how these processes contribute to intended outcomes. Considering that implementing interventions (or care strategies) into clinical practice is an integral part of KT processes, the review synthesized primary research evidence, identified implementation patterns, and examined outcomes to draw on theoretical explanations in knowledge translation for DSD care, focusing on delirium prevention and management in older people with dementia.

## Method

This review adopted a realist synthesis approach to explore the processes of knowledge translation for delirium prevention and management in people with dementia. The realist synthesis is a theory-driven method that constructs a context (C), mechanism (M) and outcome(O) configuration (CMOC) to explain causation and underlying mechanisms and understand how this combination is shaped to arrive at the outcomes through human decisions (Pawson, 2006). The review followed the steps outlined in the Realist And MEta-narative Evidence Syntheses: Evolving Standards (RAMESES) publication standards for realist synthesis (Wong et al., 2013) (Appendix 1—see online supplementary material). The synthesis was a moving back-and-forth process between steps, iteratively and reflectively, to define and redefine a program theory. The review is registered with the International Prospective Register of Systematic Reviews (PROSPERO) (Identifier no: CRD42024543493).

### Step 1: Scoping literature and establishing initial program theory

#### Define the scope of the review

The first step of a realist synthesis was to establish an initial program theory to define the review’s scope (Wong et al., 2013). An overview of the literature was conducted to explore the areas of DSD as outlined in the introduction. The information was then integrated to develop an initial program theory using the knowledge-to-action cycle (KTA) framework (Graham et al., 2006). Subsequently, five clinical stakeholders (three nurse clinicians, a medical doctor, and a clinical educator) and one family caregiver with real-world experience in caring for older people with delirium and dementia were consulted to construct the initial program theory.

#### Initial program theory

Table 1 summarizes the elements extracted from an overview of the literature and stakeholders’ inputs to build a hypothesis for an initial program theory. From the application of the knowledge-to-action framework, a common expression of a lack of practice consistency due to barriers and facilitators to prevent and manage delirium in older people with dementia is identified. The initial program theory is therefore extrapolated and established as follows:

*Clinicians caring for people with dementia with or at risk of delirium (Context) adopt strategies to address risk factors and barriers (intervention/implementation), which provide resources and guidance for individual staff to respond to (Mechanism), enabling effectiveness and sustainability in practice for preventing and managing delirium in people with dementia (Outcome).*

**Table 1:** Summary of building an initial program theory.

KTA cycle	Elements identified from the literature	CMOC configuration
Determine the Know/Do gap	Under recognition/diagnosis or diagnostic error (misattribution to dementia or depression). Absence of routine practice in prevention/management. Lack of knowledge about delirium and existing screening instruments. Lack of consensus in pharmacological treatment and non-pharmacological approaches. Complex clinical guidelines or not made aware to clinicians.	*Stakeholder 1 (nurse clinician): Delirium screening is part of the routine for older patients on admission, but it is not always completed within the first 24 hours (C).* *Stakeholder 3 (nurse clinician): It can be hard to tell the difference between delirium and dementia when they are first admitted (C).*
Adapt knowledge to local context	Predisposing/precipitating factors: older age, comorbidities, cognitive impairment/dementia, sensory impairment, trauma, surgery, infection, medications, physical restraint, and environment. Procedure-related risks: Preoperative: fasting, dehydration, hypo/hypernatremia, drug, alcohol use; Intraoperative: abdominal/cardiothoracic surgery, bleeding, procedure duration; Postoperative: pain, conditions from preoperative.	*Stakeholder 1 (nurse clinician): Staff are often overwhelmed by multiple tasks (C/M).* *Stakeholder 2 (medical doctor): Short staffing is usually one of the barriers in reality (C/M).* *Stakeholder 5 (clinical educator): People need to be aware of (delirium) and its risk factors when receiving a patient (M).*
Assess barriers/facilitators to knowledge use	Individual level: ignorance, undervalued risk factors, workload, time required for administration of screening instruments, lack of knowledge/positive attitude in delirium care for people with dementia, and absence of family/carer involvement in the care. Organisation level: work culture, low strategic/financial priority, poor leadership, lack of recognition of prevention/treatment, lack of consistency in policies and delirium treatment direction. Patient barrier: resistance to care, aggressive behaviour.	
Select, tailor, implement interventions	Prevention: avoid the use of benzodiazepines/anticholinergics, adequate analgesics and pain management, and early identification of predisposing and precipitating factors. Treatment: Non-pharmacological: education, compassionate care, potential precipitating factors, family/closed carer involvement, support/stable surroundings, orientation, nutrition and hydration. Pharmacological: low-dose haloperidol/risperidone, treating underlying causes, identifying the possibility of relapse, and adequate analgesia for pain management.	*Stakeholder 4 (nurse clinician): We use non-pharmacological interventions as first-line, then pharmacological interventions when necessary (M).* *Stakeholders 5 (clinical educator): Education is needed, and it requires ongoing effort (M).*
Monitor knowledge use	Knowledge/awareness/practice among individuals and care settings. Know/do gaps in delirium superimposed on dementia.	
Evaluate outcomes	Effectiveness of training/education/implementation on delirium superimposed on dementia Effectiveness of interventions/treatments on delirium occurrence, patient outcomes and care practices.	*Stakeholder 4 (nurse clinician): We have geriatric specialists to assess patients and evaluate treatment outcomes (M/O).* *Stakeholder 6 (family caregiver): Staying in the emergency room was not a comfortable environment for him (family member) (O).*
Sustain knowledge use	Multidomain collaboration and engagement. Maintenance of patient care quality. Delirium superimposed on dementia care policies/procedures/care standards.	

*Note.* KTA = Knowledge To Action cycle; CMOC = Contxet, Mechanism, Outcome Cofiguration; C = context; M = mechanism; O = outcome.

The scope of the initial program theory was kept broad to explore the underlying processes involved in knowledge translation of delirium care in people with dementia. The broader scope enables the identification of similarities and differences between the initial program theory and the review findings. Hence, further questions that emerged from the initial program theory are to be addressed in this review.

- What are the contextual factors and underlying mechanisms that influence the implementation outcomes of preventing and managing delirium in people with dementia?
- What are the key elements that enable knowledge to be translated into real-world practices of preventing and managing delirium in people with dementia?

### Step 2: Searching, selecting, and appraising evidence

#### Search strategy

Five databases, CINAHL, Scopus, PsycINFO, PubMed and Web Science, were searched from inception till May 2024, and an updated search was conducted in September 2025. The main search terms were obtained in consultation with an academic librarian. An iterative search was conducted using alternate words, Word Explorer, Medical Subject Headings, and Boolean operators. The search terms are shown in Table 2. The database search and search results are available in Appendix 2 (see online supplementary material).

**Table 2:** Search term.

Keyword	Main search term
Knowledge translation	“Knowledge translation” or “knowledge transfer” or “knowledge to action”
Older people	Older adults or elderly or geriatric or senior or older people or aged 65 or 65+
Delirium superimposed dementia	Delirium superimposed on dementia or delirium or dementia

#### Study selection

The database search results were imported into Covidence (Covidence systematic review software, Veritas Health Innovations, Melbourne, Australia) to streamline the study selection process. Study selection followed a three-phase process (title, abstract, and full text) in line with the inclusion/exclusion criteria. The population, concept, and context (PCC) framework (from Joanna Briggs Institute) was used to frame the review’s inclusion and exclusion criteria (Table 3). Because limited studies on DSD were found in the initial search, the inclusion criteria were expanded to include studies that, even though they did not specify the term “delirium superimposed on dementia,” still had a participant sample of older people with dementia in their intended delirium interventions. Two reviewers (YT, MG) independently screened the studies for inclusion. Disagreement was resolved during the study selection process. The final study inclusion was confirmed among the team reviewers (YT, MG, MF).

**Table 3:** Inclusion/exclusion criteria.

	Inclusion criteria	Exclusion criteria
Population	Patient: Studies on older populations aged 65 and over with a diagnosis of dementia. Clinician: Healthcare clinicians/professionals who implement interventions.	Patient: Studies on general adults under age 65 and without a diagnosis of dementia.
Concept	Studies that implement interventions to improve care practices (e.g., standard procedures, protocols, practice guidelines, educational programs, treatments, or all types of interventions. Studies that describe the experience and perception of the implementation. Studies that measure the effects and outcomes of implementation.	Studies do not have implementation elements (e.g., no implementation of an intervention or care strategy). Studies do not include people with dementia. Studies only describe the prevalence, association or testing validity of the specific tools and do not have implemented outcomes.
Context	All healthcare settings.	Intensive care units where mechanical ventilators were used
Specification	Primary research studies. All research designs and types. All-year studies. Published in English.	Unpublished sources (e.g., thesis, organisations’ internal reports). Publish other than in the English language.

#### Quality appraisal

All studies selected were appraised by three criteria of relevance, richness and rigor (Dada et al., 2023). Relevance was determined by data content relevant to the topic area, which can provide evidence for theory development/refinement/testing. Richness was determined by the density of evidence that enables the generation of CMOCs and the construction of meaningful codes to enrich theory development/refinement/testing. Rigor was determined by whether the method used to generate data was credible and trustworthy (Wong et al., 2013). Each criterion is divided into high, medium, and low based on the appraisal considerations for realist review outlined in Dada et al. (2023). The decisions were made by justifying the degree to which data aligned with the review objectives and the extent to which data contributed to theoretical insights and explanations. Rigor was assessed to determine whether the included study used validated measurements or instruments, reported procedures and used consistent methods to achieve the results. No studies were excluded based on their quality assessment.

### Step 3: Extract and synthesize evidence

Data extraction and synthesis occurred iteratively and in parallel across three levels. The first level involved deductively extracting data from each study and coding it individually into context–mechanism–outcome configurations. Considering the aim and questions of this review, each included study was read several times to identify causal relationships and outcomes within the associated contexts and mechanisms (Wong et al., 2013). The second level involved inductively extracting data from individual data codes (C/M/O) to make sense between each code and generate initial CMOCs (Gilmore et al., 2019). The third level involved abductive reasoning on the generated CMOCs and made inferences between them from individual codes. The generated CMOCs were grouped to identify their patterns and shape overarching themes. At this level, each group of CMOCs was processed logically to make inferences from the evidence. These three levels of data synthesis were conducted iteratively through a moving back-and-forth process to test the inferences against the data, ensuring that the final synthesized CMOCs are evidence-informed. The first reviewer (YT) completed the data extraction and synthesis, and the data codes and synthesized results were cross-checked by other reviewers (MG, MF). Inconsistencies between codes, generated CMOCs, and questions arising from the synthesized results were further reconfigured. Finally, the discrepancy between the initial program theory and the synthesized CMOCs was refined to define and redefine the contextual factors and underlying mechanisms of knowledge translation for preventing and managing delirium in people with dementia.

## Results

### Search results

**Figure 1:**
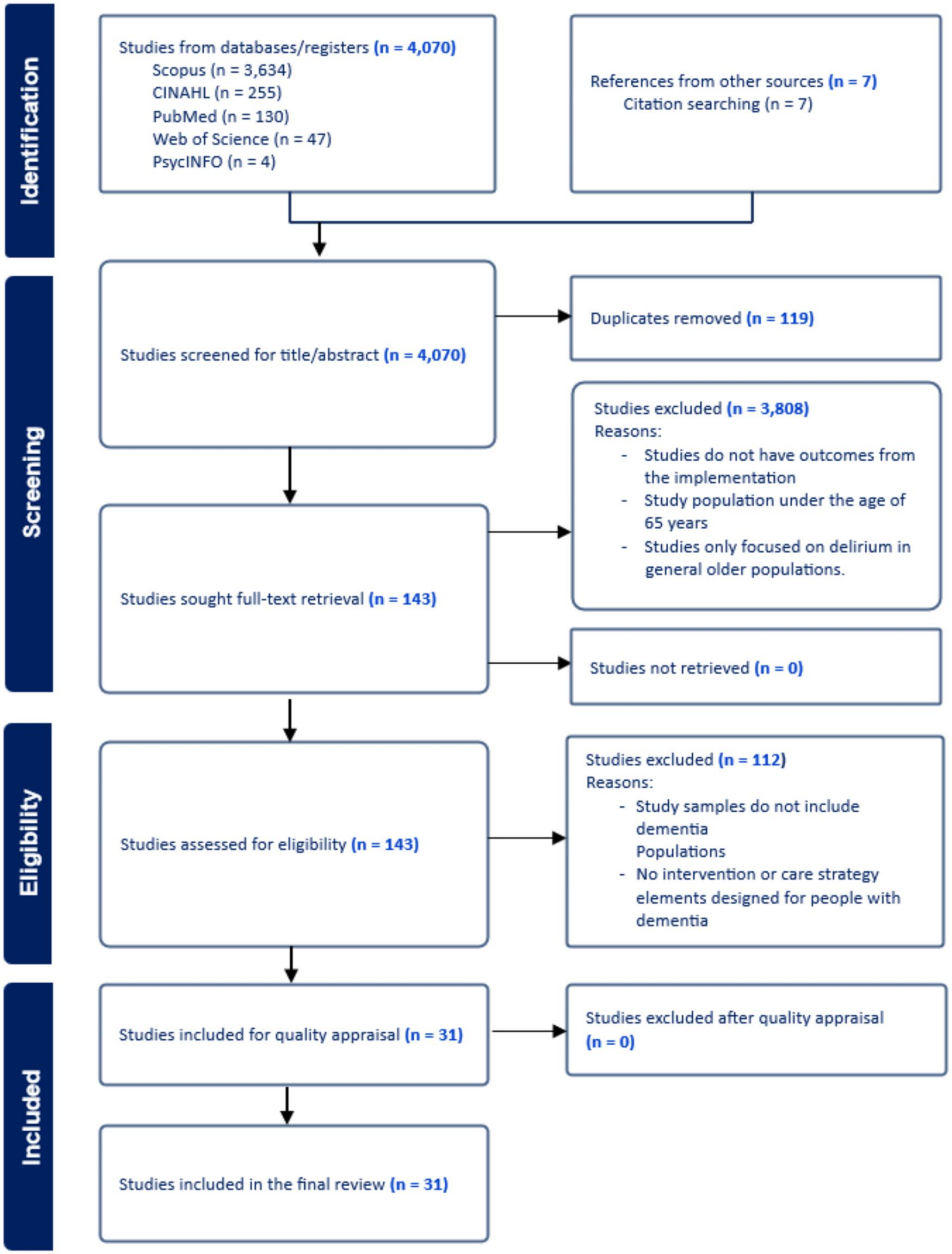
Flow chart of study selection (B&W). Figure generated from Covidence and adapted in accordance with the Preferred Reporting Items for Systematic Reviews and Meta-analysis (PRISMA) (Page et al., 2021).

A total of 4,077 references were identified from the five electronic databases, with an additional seven references identified through citation search of existing references. Of these, 4,070 references underwent title and abstract screening, and 143 studies underwent full-text screening, resulting in 31 primary studies included in this review. No studies were excluded after the quality appraisal. The excluded studies were mainly due to the absence of a dementia population in the study samples or in the delirium intervention strategies. Figure 1 shows a flow chart of the study selection in accordance with the Preferred Reporting Items for Systematic Reviews and Meta-analysis (Page et al., 2021).

### Characteristics of the included studies

The included studies encompass 16 quantitative, five qualitative, and 10 mixed-methods studies. These studies originate from various countries, including Australia (*n* = 7), Belgium (*n* = 2), Canada (*n* = 4), Italy (*n* = 1), Ireland (*n* = 1), New Zealand (*n* = 1), Singapore (*n* = 1), Switzerland (*n* = 1), South Korea (*n* = 2), United States of America (USA) (*n* = 8), and the United Kingdom (UK) (*n* = 3). They were conducted in various settings, including nursing homes (*n* = 4), hospice (*n* = 1), post-acute care (*n* = 2), conference workshops (*n* = 1), and hospitals (*n* = 23). All studies involved people with dementia, either in the study samples or within the intervention strategies. Seven studies specifically targeted older people with DSD; the others focused on cognitively impaired older populations, including dementia (*n* = 6), and general older populations with participant samples of dementia (*n* = 18). Most studies were deemed highly relevant (*n* = 22), while nine studies were of medium relevance to theory development, refinement, and testing. Six studies were rated as having high content richness, contributing to theory development, refinement, and testing; 14 were rated as moderate, and 11 as low. Thirteen studies were rated as high in methodological quality; nine as medium, and a further nine as low. Table 4 summarizes the characteristics of the included studies.

**Table 4:** Summary of included studies.

Study/year	Country	Setting	Study design	Sample	Aim	Intervention/implementation	Relevance/richness/rigor
Pozzi et al. (2020)	Italy	NH	Instruments/surveys	Patients: *n* = 22, aged older than 65 years with moderate dementia and delirium.	Evaluate the applicability of structured occupational therapy in managing patients with moderate dementia and delirium admitted to a nursing home.	Daily occupational therapy comprised functional occupation, environmental adaptation, daily living, and physical therapy for 3 weeks or until delirium resolution.	High/medium/high
Bee Gek Tay et al. (2013)	Singapore	Hospital	Prospective cohort study	Patients: *n* = 122, aged 65 years and older with delirium (of 82 patients had underlying dementia).	Examine the influence of a multicomponent delirium management program, the geriatric monitoring unit, on the functional progress of delirious older patients and the impact of underlying dementia on functional recovery.	Multicomponent delirium treatment program: early mobilization and rehabilitation, daily review of the need for intravenous drip, urinary catheter or supplementary oxygen, daily orientation, socialization, daily physiotherapy, and occupational therapy.	High/low/high
Freter et al. (2017)	Canada	Hospital	Controlled single-blind clinical trial	Patients: *n* = 283, aged 65 and older, admitted for hip fracture repair (of 77 patients with dementia)	Compare the feasibility and effectiveness of delirium-friendly preprinted post-operative orders for individuals with hip fractures, administered by regular orthopedic nurses, with routine postoperative orders.	Delirium-friendly preprinted orders: scheduled acetaminophen and lower doses/frequency of as-needed opioid analgesics; nighttime sedation option is trazodone, not benzodiazepines; domperidone for nausea, not antihistamine; urinary catheters were removed on day 2 postoperative; laxatives are scheduled; routine blood check for dehydration; low doses of haloperidol in the event of severe agitation.	High/medium/high
Lapane et al. (2011)	USA	NH	Randomised cluster trial	Patients: *n* = 3203 (2003), *n* = 3538 (2004), aged 65 and over (± 50% residents diagnosed with dementia or Alzheimer’s disease).	Determine the extent to which the use of the GRAM clinical tool would reduce the incidence of potential delirium, falls, hospitalizations potentially due to ADEs, and mortality.	Using health information technology to engage consultant pharmacists and nursing staff to identify residents at risk. GRAM reports were generated based on residents’ medications to identify resident-specific medications that potentially cause, aggravate, or contribute to delirium and fall risk. Medication monitoring care plans and flow records contained indicators of delirium and adverse drug events.	Medium/medium/high
Oberai et al. (2021)	Australia	Hospital	Prospective cohort study	Patients: *n* = 437, patients aged 65 and over admitted with hip fracture (of 129 patients with pre-existing cognitive impairment).	Implement and evaluate the effect of an intervention to prevent delirium in patients with hip fractures admitted to an acute orthopedic ward.	Included education, environmental restructuring, change champions, infographics and audit feedback reports.	Medium/low/low
Kolanowski et al. (2011)	USA	Post-acute care	Randomized control pilot study	Patients: *n* = 16, aged 65 and older, diagnosed with mild to moderate stage dementia.	Demonstrate that implementing cognitively stimulating activities is clinically feasible and potentially reduces delirium severity, duration, and functional loss in post-acute care settings.	The intervention group received cognitive stimulation delivered using recreational activities targeting cognitive domains affected by delirium: attention, orientation, memory, abstract thinking, and executive functioning.	High/low/high
Kolanowski et al. (2016) Further study by Kolanowski et al. (2011)	USA	Post-acute care	Randomized controlled trial	Patients: *n* = 283, community-dwelling, aged 65 and over with mild to moderate dementia.	Determine whether cognitively stimulating activities would reduce the duration and severity of delirium and improve cognitive and physical function to a greater extent than usual care.	Intervention group actively engage in simple activities that provide cognitive stimulation in a non-regimented way and promote processing supportive of function in the domains of attention, memory, orientation, and executive function.	High/medium/high
Deschodt et al. (2012)	Belgium	Hospital	Non-randomized Controlled trial	Patients: *n* = 171, aged 65 and over, admitted with hip fracture (of 34 patients with dementia).	Evaluate the effect of inpatient geriatric consultation teams on delirium and overall cognitive functioning in older adults with hip fractures.	The inpatient geriatric consultation team includes a geriatrician, nurse, social worker, occupational therapist and physiotherapist.	High/low/high
Hasemann et al. (2016)	Switzerland	Hospital	Pre-and post-study	Patients: *n* = 268, aged 70 and over with cognitive impairment, including dementia.	Compare the course of delirium in terms of severity and duration of delirium episodes associated with the administration of a complex delirium intervention to outcomes for a treatment-as-usual group.	DemDel consisted of 6 components: interprofessional education, screening for cognitive impairment by ward nurses, interprofessional planning of delirium prevention and management, interprofessional delirium prevention and management, screening for delirium by ward nurses, and delirium symptom management by ward nurses.	High/low/medium
Young et al. (2021) Project 3	UK	Hospital	Multicentre, pragmatic, cluster randomised controlled feasibility study	Patients: *n* = 713, aged ≥65 years, who were admitted to the study wards during the study period (of 150 patients with cognitive impairment or dementia)	Explore the potential clinical effectiveness and cost-effectiveness of the PODv2, compared with standard care, among older patients at risk of developing delirium who are admitted to hospital for emergency care.	POD has action centered on 10 risk factors associated with the development of delirium: cognitive impairment, dehydration, constipation, hypoxia, immobility, infection, multiple medications, pain, poor nutrition, sensory impairment and sleep disturbance. The implementation is through raising awareness and training of staff and volunteers.	High/low/high
O’Regan et al. (2019)	Ireland	Conference workshop	Post-survey of educational workshop	Clinicians: *n* = 62, psychiatrists.	Explore baseline attitudes, perspectives and knowledge about delirium diagnosis and management amongst psychiatrists and assess the impact of an educational intervention.	A workshop comprising four elements: 1. delirium impacts, detection, psychiatrist skill in assessment and management; 2. Neuropsychiatric syndrome, DSM diagnosis; 3. Clinical presentation, delirium-dementia interface, distinguishing from depression; 4. Non-pharmacological strategies, antipsychotic agents, and adverse effects.	High/medium/medium
Grealish et al. (2019)	Australia	Hospital	Repeated cross-sectional survey design	Clinicians: *n* = 90, registered nurses, enrolled nurses, and assistants in nursing.	To investigate the impact of the three-element delirium prevention educational program on nurses’ knowledge about delirium prevention and care over time.	The three-element education program contains 1. Online module (knowing) for delirium prevention, 2. Discussion group (meaning) about practice, 3. Simulation session (doing) depicts a patient with possible delirium.	High/medium/low
Ewens et al. (2021)	Australia	Hospital	Audit and questionnaire	Clinicians: *n* = 159, nurses, medical and allied health staff.	To evaluate the level of knowledge of delirium amongst clinicians caring for patients at high risk of developing delirium and to determine whether education can improve clinical assessment of delirium.	The education comprised a video training detailing prevalence, predisposing factors, differing presentations and associated morbidity and mortality of delirium; followed by targeted small group education in ward areas by nursing and medical educators.	High/low/low
Solberg et al. (2021)	USA	Hospital	Pre-and post-test	Clinicians: *n* = 389, nurses and patient care assistants.	To describe an education program improving recognition and attitudes towards patients experiencing delirium.	The education program contains three steps: 1. Self-directed online module, 2. Dementia stimulation experience, 3. A multi-station delirium skills fair.	High/medium/medium
Godfrey et al. (2013) Part of Young et al. (2021) Project 1	UK	Hospital	Participatory action research	Clinicians: n = 31, doctors, nurses, therapists, service managers, volunteers, caregivers. 50 hours of observation of ward practice.	Review and adapt the Hospital Elder Life Program (HELP) for use in the UK; identify strategies to support the implementation of the HELP; determine the optimum methods to deliver the HELP in routine care.	Through iterative, dialogic and reflexive approaches with multidisciplinary teams with Normalisation Process Theory to develop models of delirium prevention and delivery -the prevention of delirium system of care (POD).	Medium/medium/high
Paulson et al. (2016) Sub-study of END-DSD	USA	Hospital	Expert panel consensus	Clinicians: *n* = 32, nurses *n* = 14, ancillary staff members of the unit	Develop a delirium admission brochure for family members to aid in the prevention and early identification of delirium during hospitalisation.	Brochure content: a description of delirium, the cause of delirium, and strategies the caregivers could use to help prevent and manage delirium.	High/low/low
Yevchak et al. (2014) Sub-study of END-DSD	USA	Hospital	Descriptive, correlational study	Clinicians: *n *= 192, registered nurses, licensed practical nurses, nursing assistants, and other interdisciplinary staff	Assess barriers and facilitators to conducting nurse-led rounds as perceived by the nurse interventionist during the first 2 years of a 5-year randomized, controlled clinical trial.	Early nurse detection of delirium superimposed on dementia: nursing education, computerized decision support (embedded within the electronic health record), a designated unit delirium champion, and weekly rounding sessions with the delirium champion.	High/high/medium
Yevchak et al. (2017) Sub-study of END-DSD	USA	Hospital	Secondary analysis of rounding documentation	Clinicians: Rounding sessions of *n* = 803 patients aged 65 and over with dementia. *n* = 750, rounding sessions facilitated by nurse specialist, nurse practitioner, unit champions, and staff nurses.	Describe exploratory examples and themes of nurse-facilitated person-centered care for patients with delirium-superimposed dementia.	Weekly rounding sessions on person-centered care, for example, delirium assessment, medications, mobility, sleep, cognitive stimulation, discharge teaching, pain, communication, etc.	High/medium/medium
Kang et al. (2017)	South Korea	Hospital	Descriptive qualitative study	Clinicians: *n* = 12, registered nurses	Evaluate the effect of an educational program on registered nurses’ knowledge and attitude in delirium care for hospitalized older adults with and without dementia, and examine the program’s strengths and weaknesses from the participants’ perspectives.	Three-month educational program for delirium prevention, assessment and management of hospitalized older adults with and without dementia, using patient scenarios, role-playing, discussion, lectures and self-directed study, and provided a pocket-sized card of delirium and dementia algorithm.	High/medium/low
Pizzacalla et al. (2015)	Canada	Hospital	Pre and post-survey	Clinicians: *n* = 72, registered nurses, practical nurses, healthcare aids, occupational/physiotherapists and assistants, pharmacists, dieticians, social workers, speech pathologists, clerks and security personnel.	Describe how unit-based nursing leaders investigated, implemented and evaluated a well-established educational intervention to prepare staff to respond effectively to persons with behaviours associated with dementia and delirium.	Six one-day Workshops for introducing Gentle Persuasive Approaches.	Medium/low/low
Ervin and Moore (2014)	Australia	Hospital	Action research	Clinicians: *n* = 15, registered nurses, enrolled nurses	Explore nurses’ opinions of the implementation of the volunteer project in their work setting.	Volunteers completed a 2-day training program based on person-centered care principles and allocated shifts around patient mealtimes.	Medium/medium/low
Voyer et al. (2014)	Canada	NH	Participatory action research	Patients and clinicians: *n* = 182, patients aged over 65 with a dementia diagnosis or cognitive impairment. *n* = 19, nurses, *n* = 32, nursing aids, *n* = 48, orderlies	Examine the feasibility and acceptability of an intervention program directed at preventing delirium among cognitively impaired long-term care residents.	Delirium prevention programs (decision tree) were developed throughout three implementation cycles: training, use, feedback, and revision.	High/high/medium
Fick et al. (2011) END-DSD study	USA	Hospital	Prospective cohort study	Patients and clinicians: *n* = 15, aged 65 and older with dementia, admitted to an adult medical-surgical unit. *n* = 55, registered nurses. *n* = 9, licensed practice nurses.	Investigate the feasibility of the computerised decision support component -Early Nurse Detection of Delirium Superimposed on Dementia (END DSD).	END DSD consists of nursing education, computerised decision support through delirium screens for delirium assessment and detection and nonpharmacological management modules targeted toward nurses and facilitated with EMR, a unit champion, and a feedback mechanism to individual nurses to further facilitate assessment and management of delirium superimposed on dementia.	High/medium/high
Wand et al. (2014)	Australia	Hospital	Before-after study	Patients and clinicians: *n* = 255, patients aged 65 and older admitted to the general medical ward (of 110 patients with dementia) *n* = 77, doctors and nurses	Evaluate whether a multifaceted educational program targeting the modifiable risk factors for delirium prevented the development of delirium in hospitalized older patients on a medical ward and improved staff knowledge.	Education sessions focused on general information on delirium, prevention and management of patients with delirium.	High/high/high
Detroyer et al. (2018)	Belgium	Hospital	Before-after study	Patients and clinicians: *n* = 160 patients aged 70 and over, admitted to a geriatric ward (of 27 patients with dementia). *n* = 17, geriatric nurses	Determine the effect of a nursing e-learning tool for delirium on in-hospital prevalence, duration, severity and mortality; and geriatric nurses’ knowledge and recognition regarding delirium.	An online self-directed learning tool about delirium specifics, prevention and treatment strategies, incorporating textual information with audio-visual materials, case studies and self-assessment with feedback.	High/low/medium
Travers et al. (2018)	Australia	Hospital	Observation/chart audit	Patients and clinicians: *n* = 181, aged 65 and over with dementia and delirium. *n* = 34, experienced nurses.	Educate and empower experienced hospital nurses to lead practice changes in their wards, to improve the care of hospitalised patients with cognitive impairment by implementing best practices, particularly care processes for delirium prevention, identification and management.	Champion nurses developed and implemented ward-specific action plans with support from facilitation and education.	High/high/high
Bush et al. (2022)	Canada	Hospital	Pre-and post-study	Patients and clinicians: *n* = 40, chart audit (of 3 patients with dementia). *n* = 25, survey from physicians, pharmacists, nurses, and allied health. *n* = 10, focus groups/interviews from palliative care staff.	Adapt, implement, and evaluate a delirium guideline for the interprofessional team in an inpatient palliative care unit.	An interprofessional delirium guideline containing modules and implementation resources about features of delirium, evaluation and monitoring of a delirious patient, non-pharmacological and pharmacological management, communication, and patient/family education.	High/high/medium
Bolton and Loveard (2016)	New Zealand	Hospice	Pre-and post-survey	Clinicians and volunteers: n = 51, administrator, cleaners, allied health, care assistants, doctors, volunteers, nurses.	Development of a toolkit for interdisciplinary team members to use to improve care for people with dementia or delirium in a hospice inpatient unit.	Te Kete Marie toolkit contains: Resource role, interdisciplinary team members, education, this is me booklet, activity toolbox, environmental and reality orientation resources, core care plan guidelines, Miro-management of individual patients, Montreal cognitive assessment, consumer feedback.	Medium/low/low
Ayton et al. (2020)	Australia	Hospital	Focus groups/interviews	Clinicians and volunteers: *n* = 17, aged care registrars, hospital volunteers, consultant geriatrician, surgeon, delirium project officer, registered nurse, nurse unit manager, allied health staff, consultant physician and non-clinical staff member.	Explore the perceived barriers and enablers to implementing the Volunteer Dementia and Delirium program from the perspective of key stakeholders in a metropolitan hospital network.	The program provides a 1:1 companionship for emotional support and practical assistance to patients with dementia and delirium. Volunteers complete personal profiles of each patient and/or family caregiver to support patient-volunteer communication and guide person-centred care.	Medium/medium/low
Jeong et al. (2020)	South Korea	NH	Guideline reviews/staff interviews	Guidelines and clinicians: *n* = 6, clinical practice guidelines (CPGs). *n* = 10, managers, registered nurses, and health assistants.	Develop and implement evidence-based CPGs for delirium specific to LTC and determine the barriers perceived by healthcare professionals related to the implementation of the CPGs.	Summarized guidelines for clinical practice/implementation in preventing, detecting, and intervening delirium in older adults.	Medium/medium/low
Young et al. (2021) Project 2	UK	Hospital	Before-and-after study	Patients, clinicians, volunteers *n* = 134 patient questionnaires *n* = 80 carers questionnaires interviews with patients, carers, ward staff and volunteers. *n* = 14,968, observations of staff and volunteer activities.	Conduct a feasibility study to assess the implementation and acceptability of the prevention of delirium system of care to patients and their relatives, clinicians, support staff and volunteers and refine the content and delivery of the intervention.	The POD system of care incorporates systems and mechanisms comprising: mobilization of a staff action group; staff (and volunteer) training; review of current ward practice; examination of delirium risk factors within current ward practice; implementation of delirium practice; and the volunteer program.	High/high/high

*Note.* DemDel = Comprehensive delirium management programme; DSM = Diagnostic and Satatistical Manual of Mental Disorder; EMR = Electronic medical Record; END-DSD = Early Nurse Detection of Delirium Superimpoased on Dementia; GRAM = Geriatric Risk Assessment MedGuide; LTC = Long Term Care; NH = Nursing Home; POD = Prevention Of Delirium; ADE = Adverse Event.

### Identified program theory

Eight context–mechanism–outcome configurations under the four overarching themes had emerged from the realist synthesis of primary research as evidence-informed results in this review. Most CMOCs were constructed based on studies of medium to high (CMOCs 1, 2, 3, 6) and high (CMOCs 5, 8) methodological rigor, with two CMOCs being based on studies of low to medium-methodological quality (CMOCs 4, 7). Table 5 provides a summary of the eight synthesized context–mechanism–outcome configurations, and a list of coded CMOCs is available in Appendix 3 (see online supplementary material). The four overarching themes include time constraints, awareness, and knowledge of delirium and dementia, treatment effectiveness, and the feasibility and sustainability of implementation, reflecting persistent challenges in clinical practice. The following sections explain the eight synthesized CMOCs under these four themes to explore knowledge translation of delirium interventions for older people with dementia, at risk of or experiencing delirium, to address the review questions.

**Table 5:** Synthesized Context-Mechanism-Outcome configuration (CMOC).

Overarching theme	CMOC	Description of CMOC	Evidence studies
Time constraints	CMOC1-Integration into existing practice encourages staff readiness and increases usability and acceptability of implementation.	Patients with dementia and delirium require increased time for explanation and reinforcement of instructions (C). The barrier of time is difficult to overcome and affects the implementation of best practices across acute care settings (C). Each LTC facility has its own culture and policies (C); therefore, it is critical to lay the groundwork before implementing new tools (M). No matter how simple an intervention is, providing practical training is essential to optimise its integration (M). Small person-centred changes can be incorporated into routine clinical care and begin the movement of an institution’s culture towards (M). Creative approaches to delivering training sessions during early morning breaks and reinforcement through discussion at handovers (M). Institute mandatory delirium education training while embedding delirium screening tools in the nursing admission and shift assessment notes (M). Utilising tailored PCC helps ease the burden when staff are trained in the methods, and the environment is modified (O). Integrating the work as closely as possible into the current care context is easily accepted if it does not become an additional workload issue (O). Implementing an EMR delirium order set, the bedside nurses can activate a nonpharmacological order (M), increasing nurse confidence in treating delirium (O).	10 studies Ervin (Ervin and Moore (2014)); Fick et al. (2011); Godfrey et al. (2013); Jeong et al. (2020); Pizzacalla et al. (2015); Solberg et al. (2021); Voyer et al. (2014); Yevchak et al. (2017); Yevchak et al. (2014); Young et al. (2021)
	CMOC2-The presence of dedicated personnel improves work initiatives and supports clinical staff in addressing patient care needs in practice.	Patients with dementia and delirium require increased time for explanation and reinforcement of instructions (C). Clinical staff did not have time or capacity to provide the level of psychosocial support (C), and the presence of volunteers addressed unmet care needs for patients (M). Higher nursing ratios are essential to prevent functional decline (M). Adequate staffing levels enable the implementation of a complex intervention (M). Identification of appropriate unit champions who have a strong interest and good relationship with nursing staff is critical to the successful implementation (M). Establishment of resource roles leads to the ongoing development of the initiative and enables continued monitoring and evaluation of the quality improvement cycle (M). Dedicated time of a senior experienced nurse to lead implementation (M). Continual presence of volunteers during busy times afforded nurses the opportunity of attending to other tasks and overcoming the difficulty of competing demands in acute settings (O). Nurses’ secondment to work as facilitators effectively overcame some of the time constraints and enabled the progression of action plans (O). Volunteers can focus on the non-medical aspect of care, such as social and emotional support, particularly important for patients with no family or friend support (O). A highly engaged clinical nurse facilitator contributed to the coordination of the program (O).	12 studies Ayton et al. (2020); Bee Gek Tay et al. (2013); Bolton and Loveard (2016); Ervin (Ervin and Moore (2014)); Godfrey et al. (2013); Grealish et al. (2019); Solberg et al. (2021); Travers et al. (2018); Wand et al. (2014); Yevchak et al. (2017); Yevchak et al. (2014); Young et al. (2021)
Awareness/knowledge of delirium and dementia	CMOC3-Partnership with direct stakeholders promotes a sense of ownership and drives active participation.	Low coherence of delirium awareness across the clinical sites, delirium prevention was not perceived as meaningful (C). Staff know all the residents’ conditions; every day is the same for them (C). Staff stated they have seen little delirium here for many years (C). Use personnel in the NH to provide information and design trials to provide program applicability in daily clinical practice (M). Involvement of the clinical staff in the decision-making process helped foster a sense of ownership (M). Relationships must be formed before implementing best practices so that staff trusts and cares about the problem and is actively engaged in solutions (M). Strong interpersonal relationships among the participants from the same cultures facilitated their group work and interaction (M). Fostered creativity and problem-solving approach and active decision-making by staff in how to make change happen, contributing to staff ownership of change (O). Although we consulted different stakeholders familiar with the context of LTC settings, actual users of the DPP (nurses, nursing aids and orderlies) made a significant contribution to improvements in DPP (O). Information that was developed by the team encouraged a sense of ownership, contributing to engagement (O).	11 studies Bolton and Loveard (2016); Bush et al. (2022); Godfrey et al. (2013); Jeong et al. (2020); Kang et al. (2017); Pozzi et al. (2020); Travers et al. (2018); Voyer et al. (2014); Wand et al. (2014); Yevchak et al. (2014); Young et al. (2021)
	CMOC4-Iterative educational components facilitate clinicians’ active learning and exposure to knowledge of delirium in older people with dementia.	Lack of knowledge and education are major barriers to implementing the protocol (C). Lack of knowledge causes staff stress and workload (C). Staff’s passive attitude in between roles and misconceptions about treatment related to insufficient delirium information (C). Confidence and diagnosis of delirium with BPSD are considered especially challenging (C). Through case method, role play, discussion and non-judgemental feedback, RNs achieved a deeper understanding by relating their learning to personal experience (M). The delirium brochure provides delirium education to family members (M). Knowledge learned from e-learning modules with no other enabling strategies is less likely to influence behaviour change in practice (M). Some participants’ prior knowledge and extensive experiences of delirium informed their current practices (M). Knowledge gained from different ways of engagement through online learning, discussion and simulation attributed to nurses’ incorporation of knowledge from these experiences into their personal construction of knowledge (M). Clearly define volunteer roles and responsibilities and ensure volunteers are trained and emotionally supported (M). The training and involvement of volunteers in the care was not a static document that would be subject to review and change based on action and outcomes (M). By providing education, the absence of a sense of responsibility or role (lack of awareness) was improved, and nurses were later very engaged and interested (O). Improved knowledge and confidence reduced stress and perceived workload (O). The educational approach was a low-cost intervention (O). Baseline differences in the willingness to use antipsychotics may reflect the differing subspeciality backgrounds of respondents and likely relate to varying appreciation of research that addresses risks and benefits of pharmacotherapy in delirium (O). No difference in delirium knowledge after education, as the level of knowledge was already high (O). Following the virtual dementia tour(dementia simulation) (M), participants experienced significant changes in their sensitivity to the impact of cognitive impairment on patients’ ability to function in everyday life (O).	16 studies Ayton et al. (2020); Bolton and Loveard (2016); Bush et al. (2022); Detroyer et al. (2018); Ewens et al. (2021); Godfrey et al. (2013); Grealish et al. (2019); Jeong et al. (2020); Kang et al. (2017); O’Regan et al. (2019); Paulson et al. (2016); Pizzacalla et al. (2015); Travers et al. (2018); Wand et al. (2014); Yevchak et al. (2014); Young et al. (2021)
Treatment effectiveness	CMOC5-Acknowledging pharmacological and non-pharmacological treatments results in mixed effects in delirium onset, duration and severity.	The occupational therapy intervention was more difficult to deliver in the aggressive patients (C). Even in the specialist group, considerable variation in perception of delirium, and confidence in diagnosis and management according to different clinical presentations of delirium (C) With a software-generated report, consultant pharmacists were 4 times more likely to recommend a dose change or discontinue or monitor change in residents who triggered falls or delirium (M); thereby reducing delirium onset (O). At delirium resolution, the patients recovered almost completely from the pre-delirium cognitive and functional status (O). Early initiation of rehabilitation benefited functional recovery regardless of patients’ cognitive and functional performance at presentation (O). Participants who were treated using delirium-friendly orders had a significantly lower rate of postoperative delirium (O). E-learning tools failed to affect delirium severity and duration(O). Significant decrease in delirium rate and compliance with the screening tool, but no difference in delirium duration (O). Recreational activities targeting cognitive domains provided positive effects on delirium severity and fewer days of delirium (O). The effect of IGCT on the duration and severity of delirium was no different (O). A significant reduction of benzodiazepines as a sleeping pill and as rescue medication contributed to an improvement in delirium severity (O).	13 studies Bee Gek Tay et al. (2013); Deschodt et al. (2012); Detroyer et al. (2018); Ewens et al. (2021); Freter et al. (2017); Hasemann et al. (2016); Kolanowski et al. (2016); Kolanowski et al. (2011); Lapane et al. (2011); O’Regan et al. (2019); Oberai et al. (2021); Pozzi et al. (2020); Young et al. (2021)
Feasibility and Sustainability of implementation	CMOC6-Effective leadership, coordination and governance support promote staff adherence and contribute to successful implementation.	If it is not compulsory, the guidelines may not be used by those who are not interested (C). Non-completion of care tasks was due to no staff assuming responsibility for the tasks, a funding issue and a lack of priority effort by leadership (C). Gaining internal support and ensuring effective leadership facilitates implementation and recruitment (M). The senior team initiated regular auditing as a means to support change and remind clinicians that they are being held accountable (M). Clinical leadership is important to staff adherence for practice development (M). Stability within the project team, advanced leadership and financial support for nurses’ attendance contributed to guideline implementation (O). Non-adherence rate in one ward was caused by the head nurse, who started a discussion and criticized in front of his team (O). Engagement from the senior management level creates significant improvement (O). A highly engaged nursing unit manager contributed to the coordination of the program (O). Critical to implementation was the combined and coordinated involvement of a named individual driver at the senior level whose professional authority and vertical networks legitimised the work of implementation in the face of competing priorities (O). The successful implementation of DPP was due to effective clinician leadership to ensure proper delivery of the program (O).	9 studies Bush et al. (2022); Grealish et al. (2019); Hasemann et al. (2016); Jeong et al. (2020); Oberai et al. (2021); Travers et al. (2018); Voyer et al. (2014); Yevchak et al. (2014); Young et al. (2021)
	CMOC7-Regular feedback and addressing concerns encourage staff to maintain and sustain the implementation.	Some content is not applicable because of the fast pace, patient acuity and more invasive procedures in acute care environments (C). Reinforcement or using reminders can work at a point in time, but could also wane over time (C). Theoretical knowledge of delirium did not necessarily translate into practice (C). With a good adherence of nursing staff, it is possible to introduce delirium-friendly orders into routine care practice (M). Repetitive education is important, so we naturally keep it in mind (M). Computerised format screens prompted nurses to adhere to the care standards(M). Positive feedback from one unit spreads to other units, contributing to its sustainability in their units (O). Patients recovering from delirium motivated staff to implement the computer screen in patients exhibiting symptoms of delirium superimposed on dementia, but not enrolled in the study (O). Solicited feedback during, while and after implementation increased staff adherence to the intervention (O). The project’s impact waned following completion and is likely to continue to decline without continued emphasis on delirium as an important issue (O). The outcomes pertained only to short-term improvements in attitude, perceptions and knowledge and it is unclear whether the workshop will have any enduring effect on clinician behaviour or patient outcomes (O) The sustained level of knowledge over time following the completion of the program (O), suggests that the nurses may have developed a shared understanding of delirium prevention and care potential for social transformation throughout relations between newcomers and older timers in the educational activities (M).	11 studies Ewens et al. (2021); Fick et al. (2011); Freter et al. (2017); Grealish et al. (2019); Jeong et al. (2020); Kang et al. (2017); O’Regan et al. (2019); Pizzacalla et al. (2015); Travers et al. (2018); Wand et al. (2014); Yevchak et al. (2014)
	CMOC8-Collaborative efforts encourage group coherence and help overcome complex implementation challenges.	Helping families understand the risk factors and causes of delirium can assist the entire healthcare team in managing delirium (M). Non-pharmacological interventions described in the brochure provide a way for family members to assist in delirium care without nursing intervention (M). Each interdisciplinary member shared his/her unique knowledge and skills during rounds (M). Making changes to current practice was perceived to be a collaborative effort (C), enabling contributions from unit staff, volunteers, and family caregivers (M). Patients with delirium superimposed on dementia benefit from a multicomponent delirium treatment approach (O). Nursing staff are willing to adopt new strategies to address delirium (M), but they are likely to be more successful if the responsibility is shared and valued by other disciplines (O). Multidisciplinary/multicomponent approach contributed to positive patient functional outcomes (O). Multimodal education interventions, rather than solely relying on printed or electronic materials for guideline dissemination, led to the implementation of a novel modular guideline for the entire interprofessional team (O). An interprofessional delirium consult service (psychiatrist, geriatrician, nurse) allowed nurses to feel better prepared to understand and treat delirious patients (M, O).	14 studies Bee Gek Tay et al. (2013); Bush et al. (2022); Deschodt et al. (2012); Godfrey et al. (2013); Hasemann et al. (2016); Lapane et al. (2011); Oberai et al. (2021); Paulson et al. (2016); Solberg et al. (2021); Travers et al. (2018); Voyer et al., 2014; Wand et al., 2014; Yevchak et al., 2014; Young et al., 2021)

*Note.* BPSD = Behavioral and Psychological Symptoms of Dementia; DPP = Delirium Prevention Program; EMR = Electronic medical Record; IGCT = Inpatient Geriatric Consultation Team; LTC = Long Term care; NH = Nursing Home; PCC = Person Centred Care; RN = Registered Nurse

### Time constraints

#### Context–mechanism–outcome configuration 1

Integration into existing practice encourages staff readiness and increases usability and acceptability of implementation.

Time constraints are a common challenge that impacts the implementation of best practices in busy clinical environments (Ervin and Moore, 2014; Jeong et al., 2020; Pizzacalla et al., 2015; Yevchak et al., 2014, 2017). Every care setting has its own culture and policies that influence what is considered a priority in practice (Voyer et al., 2014). In the fast-paced care environments, time is often allocated for the most urgent tasks due to competing demands and time constraints (Ervin and Moore, 2014; Godfrey et al., 2013; Young et al., 2021). In this context, ten interventions (four targeted DSD (Ervin and Moore, 2014; Fick et al., 2011; Pizzacalla et al., 2015; Yevchak et al., 2014), one targeted cognitively impaired populations, including dementia (Voyer et al., 2014) and five for general older populations, including those with dementia (Godfrey et al., 2013; Jeong et al., 2020; Solberg et al., 2021; Yevchak et al., 2014; Young et al., 2021) adopted an integration approach to address time constraints in the intended delirium interventions. Most interventions derived from this CMOC were medium to high-methodological quality studies, implemented in hospitals and nursing homes (Jeong et al., 2020; Voyer et al., 2014).

A new care strategy is regarded as more acceptable when its delivery is closely aligned with existing practice (Godfrey et al., 2013; Voyer et al., 2014; Yevchak et al., 2017; Yevchak et al., 2014; Young et al., 2021). The most frequently mentioned integration approaches involved work adaptations through small, gradual changes incorporated into existing practice (Solberg et al., 2021; Voyer et al., 2014; Yevchak et al., 2017). Implementing small changes in providing low-cost hearing amplification devices for patients with sensory impairment enabled clinicians to better engage patients in the routine care activities, thereby increasing patients’ orientation and interaction (Yevchak et al., 2017). Dissecting the information to introduce new strategies in small sessions offered a clearer understanding and helped optimize how clinical staff integrated these strategies into daily practice (Godfrey et al., 2013; Pizzacalla et al., 2015; Voyer et al., 2014; Young et al., 2021). The short sessions held during morning breaks and handovers ensured all staff could participate, fostered team discussions, and boosted staff engagement with the new care approaches (Young et al., 2021).

In addition, incorporating a delirium education program into mandatory training increased staff uptake, improved their understanding of delirium and enhanced skills in caring for individuals with delirium and dementia (Solberg et al., 2021). Integrating a delirium order set with decision support recommendations into the hospital’s electronic record system improved adherence to delirium assessment protocols and empowered nurses to activate non-pharmacological interventions for at-risk patients (Fick et al., 2011; Solberg et al., 2021). Therefore, integrating a new care strategy into existing practices was perceived as highly acceptable by clinicians, encouraging staff’s readiness to implement it despite time constraints (Godfrey et al., 2013; Pizzacalla et al., 2015; Voyer et al., 2014; Yevchak et al., 2017; Young et al., 2021).

#### Context–mechanism–outcome configuration 2

The presence of dedicated personnel improves work initiatives and supports clinical staff in addressing patient care needs in practice.

Inadequate staffing had added to workload pressure in clinical care environments (Ayton et al., 2020; Bee Gek Tay et al., 2013; Ervin and Moore, 2014; Young et al., 2021). Older people with delirium and dementia require time for explanations and instructions, adding to staff time pressure amid multiple tasks in their work environment (Ervin and Moore, 2014). In this context, 12 interventions (two targeted DSD) (Ervin and Moore, 2014; Yevchak et al., 2017), two targeted cognitively impaired populations, including dementia (Bolton & Loveard, 2016; Travers et al., 2018), and eight for general older populations, including those with dementia (Ayton et al., 2020; Bee Gek Tay et al., 2013; Godfrey et al., 2013; Grealish et al., 2019; Solberg et al., 2021; Wand et al., 2014; Yevchak et al., 2014; Young et al., 2021)) incorporated dedicated personnel, such as delirium champions, resource persons, or trained volunteers, provided delirium-specific initiation and supported clinical staff. Most interventions derived from this CMOC were medium to high-methodological quality studies, implemented in hospitals and a hospice (Bolton & Loveard, 2016).

Dedicated personnel with a particular interest in delirium prevention and management for older people with dementia are considered critical to facilitating ongoing development, monitoring, and evaluation of implementation (Bolton & Loveard, 2016; Freter et al., 2017). When dedicated personnel were given time to act as facilitators, they enabled the progression of intended care plans and program activities (Grealish et al., 2019; Travers et al., 2018; Young et al., 2021). Therefore, the presence of delirium champions, with adequate staffing to optimize clinical practice, contributed to intended outcomes (Bee Gek Tay et al., 2013; Wand et al., 2014; Young et al., 2021).

The trained volunteers are seen as another supporting initiative in this context (Ayton et al., 2020; Ervin and Moore, 2014; Godfrey et al., 2013; Young et al., 2021). Clinical staff frequently expressed no time or capacity to provide the level of psychosocial support needed for people with delirium and dementia, despite being aware of the importance of patients’ unmet needs (Ayton et al., 2020; Ervin and Moore, 2014). The trained volunteers provided non-medical aspects of care, such as psychosocial and emotional support, especially valuable for older people without family or friend support (Ayton et al., 2020). The emotional support and comfort provided by trained volunteers assisting care activities that were not medically or nursing-oriented were valued by clinicians for their benefits in cognitive stimulation and addressing the unmet needs of older individuals with delirium and dementia (Ervin and Moore, 2014; Young et al., 2021).

### Awareness and knowledge of delirium and dementia

#### Context–mechanism–outcome configuration 3

Partnership with direct stakeholders promotes a sense of ownership and drives active participation.

Clinical staff’s lack of interest in implementing best practice for delirium prevention and management emerged from eleven interventions (one targeted DSD [Pozzi et al., 2020], two targeted cognitively impaired populations, including dementia (Bolton & Loveard, 2016; Travers et al., 2018), and eight for general older populations, including those with dementia (Bush et al., 2022; Godfrey et al., 2013; Jeong et al., 2020; Kang et al., 2017; Voyer et al., 2014; Wand et al., 2014; Yevchak et al., 2014; Young et al., 2021)). Most interventions derived from this CMOC were medium to high-methodological quality studies, implemented in hospitals and nursing homes (Jeong et al., 2020; Pozzi et al., 2020; Voyer et al., 2014).

The lack of interest in best practice has led clinical staff not to recognize the actions needed to prevent and manage delirium in people with dementia, and to treat it as a low priority in care planning (Jeong et al., 2020; Young et al., 2021). While clinical staff participation is crucial for implementing care strategies for delirium prevention and management, engagement in implementation depends on the confidence and ethos clinicians hold (Godfrey et al., 2013; Young et al., 2021). In this context, the importance of involving stakeholders in decision-making and as end-user partners was frequently emphasized (Bolton & Loveard, 2016; Bush et al., 2022; Kang et al., 2017; Travers et al., 2018; Voyer et al., 2014). Not only did engaging stakeholders inform the implementation applicability (Pozzi et al., 2020), but it also fostered a sense of ownership and built trust for problem-solving (Kang et al., 2017; Voyer et al., 2014; Yevchak et al., 2014). Moreover, the type and level of stakeholder involvement were considered crucial, particularly from the end users of the implementation. A partnership with stakeholders who are directly involved in care activities determines the extent of their sense of ownership and participation. A study focused on cognitively impaired individuals, including dementia, emphasized that, despite consulting various stakeholders familiar with long-term care settings, it was the actual users of the care strategy who made a critical difference in achieving the intended outcome (Voyer et al., 2014).

The sense of ownership from stakeholder partnership in the development of care strategies promoted strong group cohesion among clinical staff, strengthened team relationships and further facilitated their involvement in translating the new concept into practice (Kang et al., 2017; Voyer et al., 2014). As clinical staff participated in problem-solving processes, they developed a sense of ownership in the co-developed strategies, with a perceived responsibility to incorporate them into practice (Bush et al., 2022). This, in some contexts, created a shared working culture among individual staff and encouraged them to be active participants during the implementation processes (Kang et al., 2017; Yevchak et al., 2014). In addition, cultural and linguistic context differences were found to influence group cohesion on implementation. Studies from eastern countries reported generally have a collectivist culture with a preference for working together in groups to solve problems (Jeong et al., 2020; Kang et al., 2017). Therefore, establishing an informal relationship and a culturally appropriate working environment promoted strong group cohesion and encouraged staff to take ownership of the intended implementation (Kang et al., 2017).

#### Context–mechanism-–outcome configuration 4

Iterative educational components facilitate clinicians’ active learning and exposure to knowledge of delirium in older people with dementia.

A lack of awareness about delirium prevention and management in people with dementia was due to a lack of knowledge of its guidelines and protocols, or not being exposed to the needed information (Detroyer et al., 2018; Godfrey et al., 2013; Jeong et al., 2020; Young et al., 2021). Given the organizational levels of priorities, the lack of specific training on delirium and dementia was linked to staff’s passive attitudes toward their roles and responsibilities and misconceptions about delirium prevention and management in people with dementia (Jeong et al., 2020). Even for clinicians with awareness and knowledge of delirium, confidence in the accurate diagnosis of delirium, especially for people with the behavioral and psychological symptoms of dementia, was challenging (Ewens et al., 2021; O’Regan et al., 2019). In this context, 16 interventions (one targeted DSD (Pizzacalla et al., 2015), two targeted cognitively impaired populations, including dementia (Bolton & Loveard, 2016; Travers et al., 2018), and 13 for general older populations, including those with dementia (Ayton et al., 2020; Bush et al., 2022; Detroyer et al., 2018; Ewens et al., 2021; Godfrey et al., 2013; Grealish et al., 2019; Jeong et al., 2020; Kang et al., 2017; O’Regan et al., 2019; Paulson et al., 2016; Wand et al., 2014; Yevchak et al., 2014; Young et al., 2021) described ways to overcome barriers in clinical learning for delirium and dementia care. Most interventions derived from this CMOC were low to medium-methodological quality studies, implemented in hospitals, a hospice (Bolton & Loveard, 2016), a nursing home (Jeong et al., 2020), and a conference workshop (O’Regan et al., 2019).

Educational components were frequently adopted as a non-pharmacological intervention for delirium and dementia (Bolton & Loveard, 2016; Detroyer et al., 2018; Ewens et al., 2021; Grealish et al., 2019; Kang et al., 2017; O’Regan et al., 2019; Pizzacalla et al., 2015; Travers et al., 2018; Wand et al., 2014). Education or training is also a non-invasive and low-cost approach to improve clinical practice in delirium and dementia care (Wand et al., 2014; Young et al., 2021). However, regardless of which clinical setting was implemented, merely educating clinical staff is less likely to influence practice change in real-world settings (Detroyer et al., 2018; Ewens et al., 2021; O’Regan et al., 2019; Wand et al., 2014). This was reflected in those interventions that adopted educational components, but without continuous reconstruction of the individual care experience, which was deemed ineffective in making a difference in staff knowledge and confidence towards care for people with delirium and dementia (Detroyer et al., 2018; Ewens et al., 2021; O’Regan et al., 2019; Wand et al., 2014). The educational intervention for psychiatrists to manage delirium in people with dementia found that different speciality backgrounds of clinicians hold varying appreciation toward the content they received regarding the risks and benefits of pharmacotherapy, thus resulting in considerable variations in their drug prescription (O’Regan et al., 2019). An educational approach and E-learning tool were also found to be insufficient in contributing to clinical staff’s detection of delirium practice (Detroyer et al., 2018; Wand et al., 2014), particularly when detecting delirium in non-English speaking patients (Wand et al., 2014).

On the other hand, the iterative educational approach that incorporated staff’s prior knowledge and experience or used case reflection and simulation, allowed clinical staff to re-examine existing practice and gain new perspectives from their personal experience (Bush et al., 2022; Grealish et al., 2019; Kang et al., 2017; Solberg et al., 2021). In view of de-escalating delirium and dementia-associated behavioral issues, and confident in recognizing the two, clinicians valued case-based discussions for knowledge application (Pizzacalla et al., 2015; Wand et al., 2014). Thus, an iterative approach to educational components encouraged active learning and improved confidence, enabling clinical staff to apply new knowledge in their practices (Grealish et al., 2019; Kang et al., 2017; Pizzacalla et al., 2015; Solberg et al., 2021; Wand et al., 2014).

### Treatment effectiveness

#### Context–mechanism–outcome configuration 5

Acknowledging pharmacological and non-pharmacological treatment results in mixed effects on delirium onset, duration and severity.

Thirteen interventions (three targeted DSD (Kolanowski et al., 2016; Kolanowski et al., 2011; Pozzi et al., 2020), one targeted cognitively impaired populations, including dementia (Hasemann et al., 2016), and nine targeted general older populations, including those with dementia (Bee Gek Tay et al., 2013; Deschodt et al., 2012; Detroyer et al., 2018; Ewens et al., 2021; Freter et al., 2017; Lapane et al., 2011; O’Regan et al., 2019; Oberai et al., 2021; Young et al., 2021)) measured their intervention effects on delirium incidence, duration, and severity. Most interventions derived from this CMOC were high-methodological quality studies, implemented in hospitals, post-acute care facilities (Kolanowski et al., 2016, 2011), a nursing home (Lapane et al., 2011), and a conference workshop (O’Regan et al., 2019).

Non-pharmacological treatments, such as occupational therapy, physical therapy, cognitive stimulation, geriatrician consultation and clinician education, were listed as strategies to prevent and manage delirium occurrence, duration and severity (Bee Gek Tay et al., 2013; Deschodt et al., 2012; Detroyer et al., 2018; Hasemann et al., 2016; Kolanowski et al., 2011; Kolanowski et al., 2016; Oberai et al., 2021; Pozzi et al., 2020; Young et al., 2021). Implementing daily occupational and physical therapy, along with environmental modification, early mobilization and rehabilitation, positively improved delirium recovery and maintained functional performance in individuals with delirium and dementia (Bee Gek Tay et al., 2013; Pozzi et al., 2020). Implementing cognitive stimulation in older participants with DSD was found to mitigate the cognitive decline of executive functioning (Kolanowski et al., 2011; Kolanowski et al., 2016). In addition, emphasizing training on delirium risk factors has led to clinicians’ increased use of delirium screening tools (Ewens et al., 2021; Oberai et al., 2021; Young et al., 2021), while the severity and duration of delirium remained unchanged with the multidisciplinary intervention of geriatrician consultation (Deschodt et al., 2012).

Pharmacological treatments included medication monitoring, type and dose adjustments, and standardized postoperation orders (Freter et al., 2017; Hasemann et al., 2016; Lapane et al., 2011). Pharmacist-led software-generated medication monitoring has reduced delirium incidence in newly admitted residents (Lapane et al., 2011). Standardizing postoperative medication orders for drugs considered harmful to patients undergoing surgery also contributed to decreasing delirium onset in people with dementia (Freter et al., 2017). Furthermore, educational training on medication use has led to reduced use of benzodiazepines as sleeping pills or as a “delirium rescue medication,” which helped improve delirium severity in older people, including those with dementia (Hasemann et al., 2016).

Nevertheless, both pharmacological and non-pharmacological treatments presented mixed effects. Although most studies reported a decrease in delirium occurrence after the interventions (Deschodt et al., 2012; Freter et al., 2017; Hasemann et al., 2016; Lapane et al., 2011; Oberai et al., 2021; Pozzi et al., 2020; Young et al., 2021), the severity and duration of delirium were difficult to resolve through either pharmacological or non-pharmacological methods (Deschodt et al., 2012; Kolanowski et al., 2011; Oberai et al., 2021; Young et al., 2021).

### Feasibility and sustainability of implementation

#### Context–mechanism–outcome configuration 6

Effective leadership, coordination and governance support promote staff adherence and contribute to successful implementation.

Feasibility and sustainability of the implementation are quality outcomes of delirium intervention for people with dementia. One of the key elements that influences outcomes of feasibility and sustainability in clinical practice is effective leadership. Nine interventions (three targeted cognitively impaired populations, including dementia (Hasemann et al., 2016; Travers et al., 2018; Voyer et al., 2014) and six targeted general older populations, including those with dementia (Bush et al., 2022; Grealish et al., 2019; Jeong et al., 2020; Oberai et al., 2021; Yevchak et al., 2014; Young et al., 2021)) emerged with contextual factors relating to the effectiveness of leadership. Most interventions derived from this CMOC were medium to high-methodological quality studies, implemented in hospitals and nursing homes (Jeong et al., 2020; Voyer et al., 2014).

Leadership at different levels plays a crucial role in decision-making, direction, and prioritization. Lack of staff adherence to best-practice standards and delirium policies and procedures were frequently reported as funding issues or a lack of institutional priority given by the associated leadership (Jeong et al., 2020; Travers et al., 2018; Yevchak et al., 2014). In several interventions, leadership factors were reflected in characteristics and tendencies of individual leadership, accessible resources and their allocation, as well as broader organizational priorities and governance structures (Jeong et al., 2020; Travers et al., 2018; Yevchak et al., 2014; Young et al., 2021). Oftentimes, the patterns of implementation challenges are embedded in various levels of organizations, along with external regulations, where accountability and oversight from leadership remain variable, leading to clinical staff’s passive disengagement in care initiatives despite the availability of guidelines and best-practice standards (Jeong et al., 2020; Oberai et al., 2021).

Concerning this context, at the individual leadership level, the importance of gaining institutional support before initiating implementation (Voyer et al., 2014) and ensuring effective leadership buy-in to facilitate a smooth implementation process were frequently mentioned (Bush et al., 2022; Fick et al., 2011; Oberai et al., 2021; Travers et al., 2018; Yevchak et al., 2014). In the face of competing priorities, leadership provided legitimacy to protect staff time and secure financial support for education and training delivery, which is considered the main facilitator to promote successful implementation (Bush et al., 2022; Young et al., 2021). In facilitating and executing care strategies, the level of staff motivation and engagement was significantly influenced by the driving force and coordinating style of their in-line clinical leadership (Grealish et al., 2019; Hasemann et al., 2016). At the broader institutional level, organizational initiatives, policy commitment (Travers et al., 2018), governance accountability, transparency, and systemwide operations serve as an overarching force to support the stability and consistency of leadership at different levels, contributing to the effectiveness of leadership in the intended implementation (Young et al., 2021).

#### Context–mechanism–outcome configuration 7

Regular feedback and addressing concerns encourage staff to maintain and sustain implementation.

Sustaining implementation requires continuous effort. Eleven interventions (two targeted DSD (Fick et al., 2011; Pizzacalla et al., 2015), one targeted cognitively impaired populations, including dementia (Travers et al., 2018), and eight for general older populations, including those with dementia (Ewens et al., 2021; Freter et al., 2017; Grealish et al., 2019; Jeong et al., 2020; Kang et al., 2017; O’Regan et al., 2019; Wand et al., 2014; Yevchak et al., 2014)) emerged with contextual factors relating to the sustainability of implementation. Most interventions derived from this CMOC were low to medium-methodological quality studies, implemented in hospitals, a nursing home (Jeong et al., 2020), and a conference workshop (O’Regan et al., 2019).

Some interventions pertained only to short-term improvements of clinical staff’s attitudes, perceptions and knowledge. It is unclear whether education/training delivered by theoretical knowledge will have an enduring effect on clinician behaviors or patient outcomes (Ewens et al., 2021; O’Regan et al., 2019). In this context, sustainable implementation was frequently attributed to those who continuously addressed key stakeholders’ feedback (Fick et al., 2011; Pizzacalla et al., 2015) while maintaining regular education within the implementation to provide ongoing support for clinical staff in practice (Jeong et al., 2020). The DSD-specific studies found that when clinical staff discussed issues associated with their practice, they benefited from validated feelings with others, which in turn enhanced their awareness and understanding of the implementation when caring for people with delirium and dementia (Pizzacalla et al., 2015). The feedback sought during and after implementation further catalyzed staff’s motivation, leading to continued use of the required strategies in their practice (Fick et al., 2011).

The positive feedback from clinical staff who engaged in the implementation spread to other staff, encouraging social transformation to sustain intended outcomes (Grealish et al., 2019; Pizzacalla et al., 2015). Therefore, continuously emphasizing the importance of delirium prevention and management for older people, particularly for those with dementia, through regular feedback and addressing concerns, increased the likelihood of staff’s eagerness to continue applying strategies in their practice (Jeong et al., 2020; Kang et al., 2017; Pizzacalla et al., 2015). In contrast, interventions that implemented reinforcement strategies, such as reminders, were found to work at a point in time, but wore off over time when discontinued (Travers et al., 2018; Wand et al., 2014).

#### Context–mechanism–outcome configuration 8

Collaborative efforts encourage group coherence and help overcome complex implementation challenges.

Implementing intervention strategies to prevent delirium in older people with dementia and appropriately managing it is a complex process. Fourteen interventions (three targeted cognitively impaired populations, including dementia (Hasemann et al., 2016; Travers et al., 2018; Voyer et al., 2014) and eleven targeted general older populations, including those with dementia (Bee Gek Tay et al., 2013; Bush et al., 2022; Deschodt et al., 2012; Godfrey et al., 2013; Lapane et al., 2011; Oberai et al., 2021; Paulson et al., 2016; Solberg et al., 2021; Wand et al., 2014; Yevchak et al., 2014; Young et al., 2021)) emerged with contextual factors relating to collaborative efforts to address implementation challenges. Most interventions derived from this CMOC were high-methodological quality studies, implemented in hospitals and a nursing home (Lapane et al., 2011).

Factors at individual and organizational levels have impacted the feasibility and sustainability of the intended outcome. A lack of understanding of delirium and dementia at the individual level and investment in education and training at the organizational level contributed to clinical staff’s low coherence regarding delirium prevention and management practices (Godfrey et al., 2013; Young et al., 2021). Even if clinical staff were willing to adopt a new strategy introduced to them in caring for older people with delirium and dementia, they were more likely to succeed if responsibilities were shared and valued by other health disciplines (Yevchak et al., 2014). Hence, overcoming the complex challenges of delirium prevention and management in people with dementia was perceived as a collective and collaborative effort (Bush et al., 2022).

Considering this context, many interventions engaged with inter- or intra-professional teams, extended to volunteers, family caregivers and patients, to strengthen their implementation approaches (Bee Gek Tay et al., 2013; Bush et al., 2022; Deschodt et al., 2012; Godfrey et al., 2013; Paulson et al., 2016; Yevchak et al., 2014). Supporting family involvement in care activities has been reported to improve early identification of delirium in nursing assessments (Paulson et al., 2016; Wand et al., 2014). Trained volunteers working alongside nurses helped clinical staff address the emotional and psychosocial needs of older individuals with delirium and dementia (Ayton et al., 2020; Ervin and Moore, 2014; Young et al., 2021) while enhancing person-centred care (Yevchak et al., 2017).

Moreover, multi-component/multifaceted approaches often employ multiple care strategies, which have complicated implementation processes (Hasemann et al., 2016; Lapane et al., 2011; Oberai et al., 2021; Travers et al., 2018; Voyer et al., 2014; Wand et al., 2014; Young et al., 2021). The strength of a collaborative approach is to overcome the complexity of implementing a multicomponent intervention by increasing collective coherence among healthcare professionals. This approach, in turn, eased the complexity of implementation processes (Bush et al., 2022; Young et al., 2021). Specifically, interprofessional participation in care activities created a sense of community among clinical staff (Bush et al., 2022) and shaped group coherence to address the need for change in the existing work patterns (Young et al., 2021). Nurses felt better prepared to care for individuals with delirium and dementia when an interprofessional consult service was established in their clinical support network (Solberg et al., 2021). A collaborative effort was further found to help overcome multifactorial causes of delirium in people with dementia, with different health disciplines addressing multiple risk factors such as nutrition, dehydration, ambulation, and medications (Bee Gek Tay et al., 2013; Bush et al., 2022; Deschodt et al., 2012; Lapane et al., 2011; Yevchak et al., 2014).

### Refined program theory

**Figure 2:**
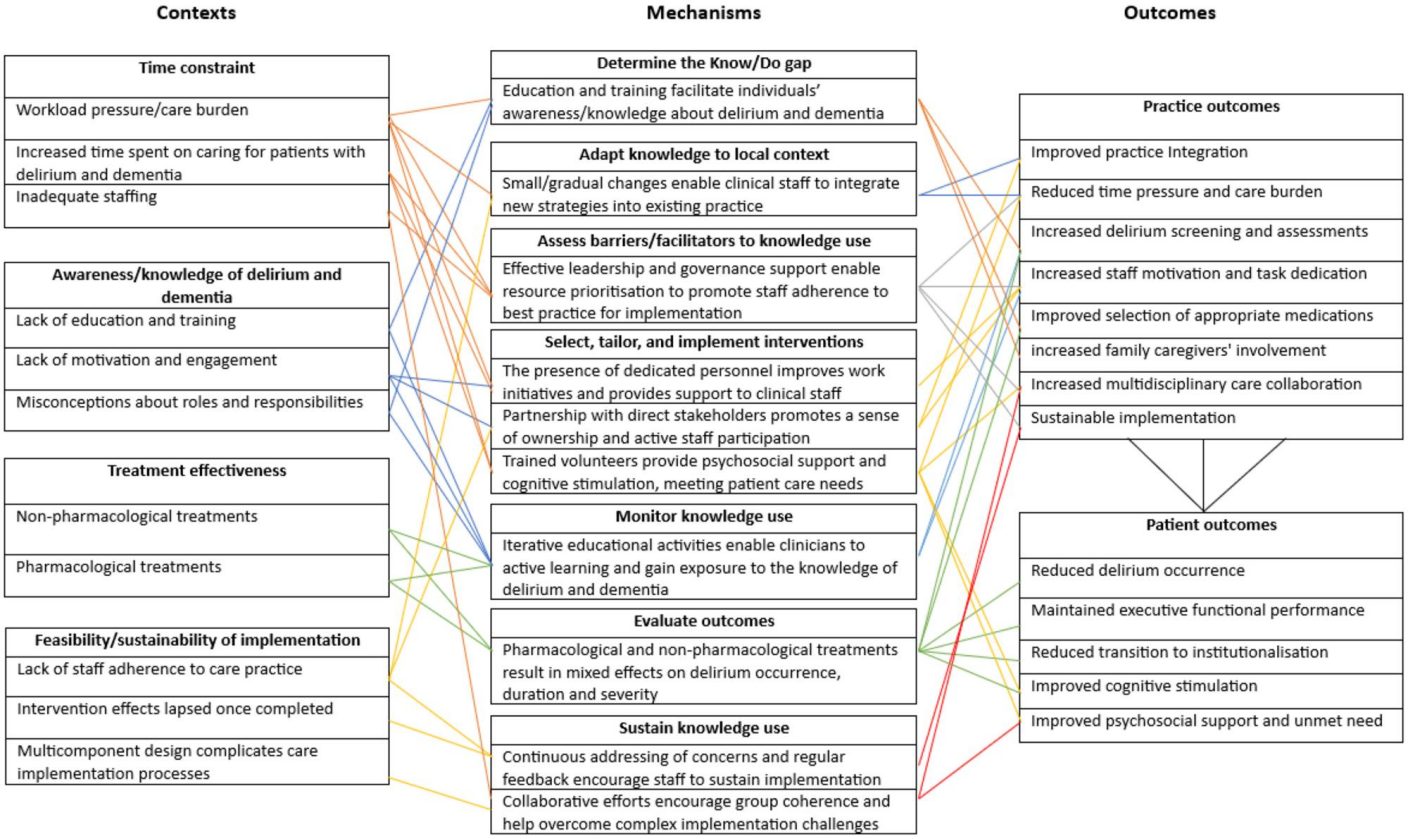
Refined program theory (B&W).

Following the eight synthesized CMOCs derived from the included primary research evidence, the program theory was refined to define and redefine the contextual factors and underlying mechanisms of knowledge translation for delirium prevention and management in people with dementia. The initial program theory hypothesized that, when clinicians were provided with and adopted delirium interventions, they would be able to effectively implement strategies to prevent and manage delirium in people with dementia to achieve the intended outcome. The refined program theory provides an expanded explanation of the contextual factors and underlying mechanisms that influence implementation outcomes and identifies key elements that would enable knowledge translation in delirium care for people with dementia. Within the KTA framework, the eight synthesized CMOCs were realigned at each step within the cycle. Because the KTA framework is an active, circular process, each CMOC within the KTA cycle is considered to interact with and influence the others, thereby establishing iterative processes for translating knowledge into practice when caring for older people with delirium and dementia. The refined program theory is shown in Figure 2.

## Discussion

### Summary of findings

This review presents the first realist synthesis of knowledge translation for delirium care in people with dementia. Using the knowledge-to-action cycle, the review explores how, why, and under what circumstances knowledge can be translated into complex clinical practice. Under the four overarching themes of time constraints, awareness and knowledge of delirium and dementia, treatment effectiveness, and the feasibility and sustainability of implementation, the key elements that enable knowledge translation when implementing delirium intervention in people with dementia were identified. These key elements, derived from the eight synthesized CMOCs, include integration into existing practice, dedicated personnel, partnership with direct stakeholders, iterative education and training, effective leadership, acknowledgment of pharmacological and non-pharmacological mixed effects, and ongoing attention to feedback and collaborative efforts. These CMOCs were derived from different clinical contexts, shared barriers and facilitators to translating knowledge/interventions into practice. Almost every CMOC was based on hospital contexts, whereas nursing home contexts accounted for more studies in CMOC 3. Medium to high (CMOCs 1, 2, 3, 6) and high (CMOCs 5 and 8) methodologically quality-ranked evidence serve as quality indicators for future application of delirium interventions and implementations.

Specifically, the element of group coherence that emerged from collaborative efforts in CMOC 8, influenced by group cohesion from cultural and linguistic contexts in CMOC 3, is critical to fostering a sense of community and ownership in healthcare implementation. The element of effective leadership that emerged in CMOC 6 reflects broader systemwide dynamics, organizational priorities, limited training and funding models, as well as the diffusion of responsibility and oversight of best-practice recommendations. This suggests that the prevention and management of delirium in people with dementia were frequently overlooked or deprioritized within healthcare systems despite established standards. Institutional engagement and accountability are as critical as frontline leadership in implementation and in sustaining active knowledge translation for preventing and managing delirium in people with dementia.

The key elements identified from the eight CMOCs distinguish the refined program theory from the initial program theory, as they expand previous assumptions and offer a theoretical explanation of what enables knowledge translation, leading to the expected outcomes in real-world practice. In other words, translating knowledge into practice of delirium prevention and management for people with dementia is likely to occur if these key elements are met together. These synthesized findings in the refined program theory provide an evidence-informed synthesis to deepen understanding of how clinical practices can be better aligned with acquired knowledge in complex and dynamic knowledge translation processes for delirium and dementia to achieve intended outcomes.

### Strengths and limitations

This review goes beyond the previously identified barriers and facilitators in the context of delirium prevention and management in older people with dementia. It explores how knowledge can be translated into care practices. Very few reviews have systematically gathered evidence for DSD. This review presents the first realist synthesis of available primary research evidence on delirium prevention and management in people with dementia. The application of the knowledge-to-action cycle framework strengthens the depiction of knowledge transfer processes, explaining how underlying mechanisms can lead to intended outcomes in the given context. Most constructed CMOCs were based on studies of medium- and high-methodological quality, indicating sufficient data quality for evidence synthesis and interpretation. The methodological quality ranking also hierarchizes the synthesized evidence among the individual CMOCs for future application.

Nevertheless, the review was limited by the amount of primary research evidence available for DSD, as most literature generalized their delirium interventions to an older population, either with the inclusion of people with or without dementia. The limited evidence for DSD has impacted the review to provide a targeted population for synthesis. Due to this limitation, the inclusion criteria were extended to include studies involving older participants with dementia, even though they did not specify the term “DSD.” Therefore, although evidence specifically for DSD populations was limited in this review, all included primary studies contained dementia populations in their intended delirium interventions for implementation. This approach has ensured that the interpretation and conclusion from the synthesized evidence apply to older people living with dementia.

### Comparison with existing literature

The complexity of delirium superimposed on people with dementia, owing to the multifactorial nature of symptoms and prognosis of delirium and dementia, complicates the best practice in care implementation (Morandi & Bellelli, 2020). Previous reviews have identified barriers and facilitators for delirium prevention and management in the older population (Dewing, 2013; Morandi et al., 2015; Schnitker et al., 2020; Siddiqi et al., 2016) and discussed the effectiveness of pharmacological and non-pharmacological interventions in various settings (Eckstein & Burkhardt, 2019; Lay et al., 2024; Lee et al., 2020; Pozzi et al., 2023; Yanamadala et al., 2013). Understanding the epidemiology and aetiology of delirium and identifying practice barriers is essential for preventing and managing delirium in people with dementia (Morandi & Bellelli, 2020; Nitchingham & Caplan, 2021). Discrepancies in translating individual understandings and knowledge into sustainable care practices are an ongoing effort in healthcare systems (Carpenter et al., 2021). Given the persistent challenge of knowledge translation, there is a paucity of literature that provides theory-based explanations and addresses complex knowledge-to-action gaps in the context of DSD. The review supports existing literature on delirium interventions for older populations, including people with dementia (Filiatreault et al., 2023; Hosie et al., 2017). Therefore, moving beyond the current evidence to explore how, why, and under what circumstances the knowledge can be translated into care practices. The refined program theory shapes understanding from the synthesis of primary research evidence, contributing to the current literature in knowledge translation for delirium care in people with dementia.

### Implications and recommendations for future research and practice

This realist review synthesized primary research evidence and explored knowledge translation for delirium care in people with dementia. The key elements excerpted from the refined program theory provide further implications and recommendations for future research and practices, as listed below:

1. Given that time constraints are an inevitable challenge in most healthcare settings, integrating new strategies into existing practice and having the presence of dedicated personnel would promote work initiatives for clinical staff and provide them with support in the implementation.
2. Individual awareness, knowledge, and perception about delirium and dementia, as well as cultural and linguistic context differences, could vary the personal motivation and participation in practice. Partnering with direct end-users in developing strategies can imbue a sense of ownership and empower active participation in best practices of delirium care for people with dementia.
3. Providing education and training alone is insufficient to support clinical staff in translating knowledge into practice. It requires clinicians to continue reconstructing individual experiences, incorporate prior knowledge and care experience, and re-examine their existing practice with newly gained perspectives.
4. With mixed effects presented in pharmacological and non-pharmacological treatments, the duration and severity of delirium in people with dementia can only be reduced to some extent. Thus, effective implementations should acknowledge the progression of delirium once it has occurred. This would empower clinical staff to maintain effective preventative measures for people with a high risk of acquiring delirium.
5. Effective leadership is crucial for successful implementation. Implementing delirium interventions for people with dementia not only requires leadership buy-in and oversight, but also demands governance support, organizational accountability, and institutional prioritization of care practice. Alignment of implementation strategies across organizational polices, accreditation standards, and regulatory frameworks may actively strengthen evidence-based practice, adoption and sustainability of implementation beyond the influence of individual leadership.
6. Sustainable implementation is achievable by continuously addressing clinical staff concerns and feedback, and emphasizing the importance of delirium prevention and management in people with dementia.
7. Current evidence indicates that multicomponent approaches have a better effect on delirium prevention and management. Although multicomponent approaches are considered beneficial for addressing the complexity of DSD, they add complexity to real-world practices. Thus, a collaborative effort involving multidisciplinary clinicians, patients, family, and trained volunteers is beneficial for increasing collective coherence, sharing responsibilities and easing the complexity of care practices for people with dementia and/or at risk of delirium.
8. Limited research on DSD-specific studies is found in this review. The scarcity of primary research on this particular older population has impacted the targeted evidence that can be used to improve delirium prevention and management for people living with dementia. More research targeting interventions for DSD is needed.
9. The refined program theory derived from evidence-informed synthesis identifies key elements in knowledge translation. Further testing of the program theory is needed to examine its application in real-world practices and strengthen the evidence base in supporting delirium prevention and management for people with dementia.

## Conclusion

This realist review synthesized primary research evidence on delirium prevention and management in people with dementia. While a few interventions were available for DSD, focusing solely on dementia populations in the synthesized studies provides a foundation for future application of delirium interventions to this older group. Findings suggest that knowledge translation is influenced by various contextual factors, including time constraints, awareness and knowledge of delirium and dementia, treatment effectiveness, and the feasibility and sustainability of implementation. Key mechanisms supporting successful implementation include integrating into existing practice, dedicated personnel, partnership with direct stakeholders, iterative education and training, effective leadership, acknowledging both pharmacological and non-pharmacological effects, and ongoing attention to feedback and collaborative efforts. Overall, the current synthesis indicates that successful knowledge translation for delirium care in people with dementia depends less on a single mechanism and more on adapting to different contexts to achieve sustainable outcomes. The constructed CMOCs offer insights beyond the barriers and facilitators previously identified in the literature. With synthesized findings from quality-ranked evidence, this review refined a program theory grounded in primary research to provide new insights and recommendations for future implementation and practice. Moving forward, future research should continue to prioritize theory testing and context-specific studies to strengthen evidence base and support knowledge translation into clinical practice for delirium prevention and management in people with dementia.

## Supplementary Material

gnag071_Supplementary_Data

## Data Availability

All data generated or analyzed during this study are included in this published article and its supplementary file.

## Review protocol registration

The review is registered with the International Prospective Register of Systematic Reviews (PROSPERO) (Identifier no: CRD42024543493).
