# Supplementary material for: Knowledge translation for delirium superimposed on dementia care: a realist review
Source: Gerontologist. 2026 Apr 29;66(6):gnag071. doi: 10.1093/geront/gnag071 (PMC13200739; doi:10.1093/geront/gnag071)
Supplement: gnag071_Supplementary_Data [file gnag071_supplementary_data.pdf]

Title: Knowledge translation for delirium superimposed on dementia care: A realist review

Authors:

1. Yvette I-Pei TSAI, School of Nursing & Midwifery, University of Newcastle, University Drive, Callaghan NSW 2308 Australia. Orcid:0000-0003-1549-6902
2. Minah Amor GAVIOLA, School of Nursing & Midwifery, University of Newcastle, University Drive, Callaghan NSW 2308 Australia. Orcid:0000-0001-9685-9642
3. Mathieu FIGEYS, School of Nursing & Midwifery, University of Newcastle, University Drive, Callaghan NSW 2308 Australia. Orcid:0000-0002-7101-689X

Corresponding author: Yvette I-Pei Tsai, Yvette.Tsai@newcastle.edu.au

This work was conducted at the University of Newcastle, University Drive, Callaghan, NSW 2308, Australia.

Review protocol registration:

The review is registered with the International Prospective Register of Systematic Reviews (PROSPERO) (Identifier no: CRD42024543493).

Acknowledgements: We would like to thank the University academic librarian, Nicole Faull-Brown, for assisting with keywords and search terms during the database search.

Conflict of interest: The authors have no conflict of interest to declare.

Funding: This research did not receive any specific grant from funding agencies in the public, commercial, or not-for-profit sectors.

Data availability statement: All data generated or analysed during this study are included in this published article and its supplementary file.

Title: Knowledge translation for delirium superimposed on dementia care: A realist review

Appendix 1. RAMESS publication standards for realist synthesis

TITLE			
1		In the title, identify the document as a realist synthesis or review	√
ABSTRACT			
2		While acknowledging publication requirements and house style, abstracts should ideally contain brief details of: the study's background, review question or objectives; search strategy; methods of selection, appraisal, analysis and synthesis of sources; main results; and implications for practice.	√
INTRODUCTION			
3	Rationale for review	Explain why the review is needed and what it is likely to contribute to existing understanding of the topic area.	√
4	Objectives and focus of review	State the objective(s) of the review and/or the review question(s). Define and provide a rationale for the focus of the review.	√
METHODS			
5	Changes in the review process	Any changes made to the review process that was initially planned should be briefly described and justified.	√
6	Rationale for using realist synthesis	Explain why realist synthesis was considered the most appropriate method to use.	√

TITLE			
7	Scoping the literature	Describe and justify the initial process of exploratory scoping of the literature.	√
8	Searching processes	While considering specific requirements of the journal or other publication outlet, state and provide a rationale for how the iterative searching was done. Provide details on all the sources accessed for information in the review. Where searching in electronic databases has taken place, the details should include, for example, name of database, search terms, dates of coverage and date last searched. If individuals familiar with the relevant literature and/or topic area were contacted, indicate how they were identified and selected.	√
9	Selection and appraisal of documents	Explain how judgements were made about including and excluding data from documents, and justify these.	√
10	Data extraction	Describe and explain which data or information were extracted from the included documents and justify this selection.	√
11	Analysis and synthesis processes	Describe the analysis and synthesis processes in detail. This section should include information on the constructs analyzed and describe the analytic process.	√
RESULTS			
12	Document flow diagram	Provide details on the number of documents assessed for eligibility and included in the review with reasons for exclusion at each stage as well as an indication of their source of origin (for example, from searching databases, reference lists and so on). You may consider using the example templates (which are likely to need modification to suit the data) that are provided.	√

TITLE			
13	Document characteristics	Provide information on the characteristics of the documents included in the review.	√
14	Main findings	Present the key findings with a specific focus on theory building and testing.	√
DISCUSSION			
15	Summary of findings	Summarize the main findings, taking into account the review's objective(s), research question(s), focus and intended audience(s).	√
16	Strengths, limitations and future research directions	Discuss both the strengths of the review and its limitations. These should include (but need not be restricted to) (a) consideration of all the steps in the review process and (b) comment on the overall strength of evidence supporting the explanatory insights which emerged.<br>The limitations identified may point to areas where further work is needed.	√
17	Comparison with existing literature	Where applicable, compare and contrast the review's findings with the existing literature (for example, other reviews) on the same topic.	√
18	Conclusion and recommendations	List the main implications of the findings and place these in the context of other relevant literature. If appropriate, offer recommendations for policy and practice.	√
19	Funding	Provide details of funding source (if any) for the review, the role played by the funder (if any) and any conflicts of interests of the reviewers.	NA

Appendix 2. Search Strategy

CINAHL Complete 7/5/2024

EBSCOhost

Tue, May 7, 2024 7:16:39 PM

#	Query	Limiters/Expanders	Last Run Via	Results
S10	("delirium superimposed on dementia") AND (S2 AND S7 AND S8)	Expanders - Apply equivalent subjects<br>Search modes - SmartText Searching	Interface - EBSCOhost Research Databases<br>Search Screen - Advanced Search<br>Database - CINAHL Complete	255
S9	("delirium superimposed on dementia") AND (S2 AND S7 AND S8)	Expanders - Apply equivalent subjects<br>Search modes - Boolean/Phrase	Interface - EBSCOhost Research Databases<br>Search Screen - Advanced Search<br>Database - CINAHL Complete	0
S8	"delirium superimposed on dementia"	Expanders - Apply equivalent subjects<br>Search modes - Boolean/Phrase	Interface - EBSCOhost Research Databases<br>Search Screen - Advanced Search<br>Database - CINAHL Complete	100
S7	"older adults OR older people OR older person OR elderly OR senior"	Expanders - Apply equivalent subjects<br>Search modes - SmartText Searching	Interface - EBSCOhost Research Databases<br>Search Screen - Advanced Search<br>Database - CINAHL Complete	149,413
S6	"older adults OR older people OR older person OR elderly OR senior"	Expanders - Apply equivalent subjects<br>Search modes - Boolean/Phrase	Interface - EBSCOhost Research Databases<br>Search Screen - Advanced Search<br>Database - CINAHL Complete	0
S5	"knowledge translation OR knowledge transfer OR knowledge to action" OR "older adults OR older people OR older person OR elderly OR senior"	Expanders - Apply equivalent subjects<br>Search modes - SmartText Searching	Interface - EBSCOhost Research Databases<br>Search Screen - Advanced Search<br>Database - CINAHL Complete	22,864
S4	"knowledge translation OR knowledge transfer OR knowledge to action" OR "older adults OR older people OR older person OR elderly OR senior"	Expanders - Apply equivalent subjects<br>Search modes - Boolean/Phrase	Interface - EBSCOhost Research Databases<br>Search Screen - Advanced Search<br>Database - CINAHL Complete	0
S3	older adults OR older people OR older person OR elderly OR senior	Expanders - Apply equivalent subjects<br>Search modes - Boolean/Phrase	Interface - EBSCOhost Research Databases<br>Search Screen - Advanced Search<br>Database - CINAHL Complete	251,940
S2	"knowledge translation OR knowledge transfer OR knowledge to action"	Expanders - Apply equivalent subjects<br>Search modes - SmartText Searching	Interface - EBSCOhost Research Databases<br>Search Screen - Advanced Search<br>Database - CINAHL Complete	22,788
S1	"knowledge translation OR knowledge transfer OR knowledge to action"	Expanders - Apply equivalent subjects<br>Search modes - Boolean/Phrase	Interface - EBSCOhost Research Databases<br>Search Screen - Advanced Search<br>Database - CINAHL Complete	0

PsycINFO <1806 to April Week 3 2024>

1 ('knowledge translation' or 'knowledge transfer' or 'knowledge to action').mp. [mp=title, abstract, heading word, table of contents, key concepts, original title, tests & measures, mesh word] 6485

3 (older adults or elderly or geriatric or senior or older people or aged 65 or 65+).mp. [mp=title, abstract, heading word, table of contents, key concepts, original title, tests & measures, mesh word] 234540

4 (delirium superimposed on dementia or delirium).mp. [mp=title, abstract, heading word, table of contents, key concepts, original title, tests & measures, mesh word] 8810

5 1 and 3 and 4 4

PubMed 7/5/2024

Search	Actions	Details	Query	Results	Time
#1			Search: ((((((((knowledge translation) OR (knowledge transfer)) OR (knowledge to action)) AND (older adults)) OR (older people)) OR (older person)) OR (senior)) OR (elderly)) AND (delirium superimposed on dementia))	130	04:50:00

Scopus 7/5/2024

((ALL (delirium AND superimposed AND on AND dementia) OR ALL (delirium))) AND (((ALL (older AND adults) OR ALL (older AND people) OR ALL (older AND person) OR ALL (elderly) OR ALL (senior))) AND ((ALL (knowledge AND translation) OR ALL (knowledge AND transfer) OR ALL (knowledge AND to AND action))))

Result:3634

Web Science 24/4/2024

47 suggested results from the Web of Science Core Collection for:

knowledge translation' or 'knowledge transfer' or 'knowledge to action' (All Fields) and older adults or elderly or geriatric or senior or older people or aged 65 or 65+ (All Fields) and delirium superimposed on dementia or delirium (All Fields)

Appendix 3. Coded context-mechanism-outcome (CMOC)

Initial CMOC	CMO Code	Evidence studies
Daily occupational therapy involving environmental modification, functional tasks and early mobilisation was effective for patients to recover from DSD.<br><br>Patients with aggression made it difficult to deliver the intervention.<br><br>Use NH personnel providing applicability to the OT program	The intervention was more difficult to deliver to the aggressive patients. (C, O)<br>At delirium resolution, the patients recovered almost completely from the pre-delirium cognitive and functional status. (O)<br>Effective OT programs include environmental assessment, problem-solving strategies, interactive caregiver education, and training. (M)<br>Considering that the mental status evaluation and motor fluctuations are important features in the monitoring of DSD. (M)<br>Another key point is that we used the personnel available in the NH to provide information to design future trials, considering the applicability of a structured OT protocol in daily clinical practice. (M)<br>The OT can act as a facilitator of engagement in daily life activities, enabling the maintenance of a meaningful routine by implementing the best practice for managing delirium (M)	(Pozzi et al., 2020)
Develop stakeholder-researcher collaborative partnerships and a closer connection to end-users, optimising successful implementation.<br><br>The participatory approach gave rise to a sense of ownership.<br><br>Gaining internal support and ensuring effective leadership facilitates implementation and recruitment.	No matter how simple an intervention, providing practical training is essential to optimise its integration. (C)<br>Although we consulted different stakeholders familiar with the context of LTC settings, actual users of the DPP (nurses, Nursing aids and orderlies) made a significant contribution to improvements in the DPP. (M)<br>Involvement of the clinical staff in the decision-making process helped foster a sense of ownership of the DPP. (M)<br>Each LTC facility has its own internal culture and policies. Therefore, it is crucial to lay the groundwork before the implementation of new tools. (C)<br>It is important to gain internal support for using the DPP tools, not only at the administrative level but also from the clinical staff. (C)	(Voyer et al., 2014)
Early recognition of delirium, actively addressing all predisposing and precipitating factors, and emphasising rehabilitation with a multidisciplinary/multicomponent approach contributed to positive patient functional outcomes.<br><br>Early initiation of rehabilitation benefited functional recovery regardless of patients' cognitive and functional performance at presentation.	Avoiding physical restraint, a higher nursing ratio, and early mobilisation are essential to prevent functional decline. (M)<br>Patients with dementia didn't take longer to recover from delirium, nor did they require a longer duration to make similar functional gains. (C)<br>Patients with DSD benefit from a multicomponent delirium treatment program to the same extent as delirious patients without dementia. (M)	(Bee Gek Tay et al., 2013)
An iterative approach to developing an educational brochure improved staff recommendations for continued usage.<br><br>Family members can learn to use non-pharmacological interventions to improve delirium symptoms in hospitalised older adults.	Helping families understand the risk factors and causes of delirium can assist the entire healthcare team in managing delirium(M).<br>The iterative process provided an easy-to-read educational brochure for family caregivers. (M)<br>High recommendation for future use by nursing staff, but lacked current dissemination. (O)<br>Lack of evaluation from family caregivers. (C)<br>Non-pharmacological interventions described in the brochure provide a way for family members to assist in delirium care without direct nursing supervision. (M)	(Paulson et al., 2016)
The barrier of time is difficult to overcome and affects the implementation of best practices across acute care settings.<br>Nurses must be given the time, resources, and support from	Three major barriers: being busy on the unit, lack of awareness, and no study patients for implementing delirium rounds. (C)<br>By providing education, the absence of a sense of responsibility or role regarding delirium (lack of awareness) was improved, and nurses were later very engaged and interested. (M)	(Yevchak et al., 2013)

Initial CMOC	CMO Code	Evidence studies
institutional leadership in response to increasing nursing tasks or responsibilities for delirium care.<br><br>Integrating the work as closely as possible into the current care context may be more easily accepted if it does not become an additional workload issue.<br><br>Nursing staff are willing to adopt new strategies to address delirium, but they are likely to be more successful if the responsibility is shared and valued by other disciplines.<br><br>Including interdisciplinary colleagues improves the outcomes of nursing rounds.<br>Relationships must be formed before implementing best practices in the hospital setting so that staff trust and care about the problem and are actively engaged in solutions.<br><br>Identification of appropriate unit champions who have a strong interest in gerontology and good relationships with nursing staff and administrative leadership is critical to the successful implementation of nursing-led rounds.	Facilitators: interdisciplinary nature, prior connections to the unit, intrinsic motivation and engagement. (C)<br>Each interdisciplinary member shared his/her unique knowledge and skills during rounds. (M)<br>Patients recovering from delirium motivated staff to round. (M, O)<br>With intrinsic motivation and engagement, nurses implement the computer screens in patients exhibiting symptoms of dementia/delirium but not enrolled in the study. (M). Individual, cultural and administrative factors affect translating delirium best practice into everyday clinical practice. (C, M)	
Key informants' involvement ensured the educational programme met the readiness for adult learning needs, which stimulated participating RNs' interest and ability to learn.<br><br>Using adult learning methods facilitated active learning, which enables participants to apply knowledge to new situations and in practice.<br><br>Strong interpersonal relationships among the participants from the same cultures facilitated their group work and interaction.<br><br>Topics need to reflect the participants' learning needs and concerns in clinical practice.	Key informants and medical ward managers at the hospital site provided support and relevant information, which allowed the programme to be tailored to the needs of RNs. (M)<br>Through case method, role play, discussion, selected presentation, and nonjudgmental feedback, RNs achieved a deeper understanding by relating their learning to personal experience, re-examining existing practice, gaining new knowledge and applying the knowledge in practice. (M, O)<br>An informal relationship among the participants created strong group cohesion, which promoted a comfortable and supportive environment. (M)<br>A collectivist culture among Asian countries has a preference for working together in groups to solve problems. (C). The programme encourages group learning, which is particularly applicable to this context. (C, M)<br>Participants suggested the need for elaborating on delirium-causing drugs to be more relevant to clinical practice. (C)<br>A need for hospital management to develop comprehensive policies and protocols. (C)<br>Lack of knowledge and education as major barrier to implementing that protocol. (C, M)	(Kang et al., 2017)

Initial CMOC	CMO Code	Evidence studies
Educational support is a necessary precursor for nurses to incorporate protocols and policies in practice.		
Listening to staff concerns and addressing them with examples helps overcome implementation challenges.<br><br>Positive feedback from one unit spreads to other units of the hospital, which contributes to its sustainability in those units and advocates for the spread of GPA throughout the facility.	Participants challenged some content as not being applicable because of the faster pace, patient acuity and more invasive procedures in an acute care environment. (C) --Coaches encouraged participants to consider the time required when behaviour escalates to the point of being self-protective. (M)<br>Participants pointed out GPA didn't always work, and sometimes patients still escalate(C)--example was given in comparison that CPR is not always successful, yet is an intervention consistently attempted when indicated(C, O)<br>Staff were asking each other about their success, which benefits staff by being able to discuss issues associated with the intervention in their daily work. (M, O)<br>Staff perceived that those validating feelings, using distraction and reflection, were effective in preventing and de-escalating the behaviours of patients with delirium and dementia. (M, O)<br>Staff perceived fewer code whites (violent), and restraints were used. (O)	(Pizzacalla et al., 2015)
Individuals with dementia benefited from preventing postoperative delirium by implementing Delirium-friendly PPOs.<br><br>With good adherence of nursing staff, it is possible to introduce delirium-friendly PPOs into routine care practice.<br><br>There is no power to determine which PPOs were associated with the reduction of delirium; multifactorial approaches are more effective.	Intervention group participants who were treated using delirium-friendly orders had a significantly lower rate of postoperative delirium. (O)<br>Intervention has a stronger effect on participants with preexisting dementia. (O)<br>Pain control using regular acetaminophen has been shown to reduce postoperative opioid consumption. (O); opioids can contribute to postoperative delirium. (C, O)<br>Episodes of postoperative delirium were associated with longer stays, greater risk of death and nursing home placement. (O)	(Freter et al., 2017)
Volunteers' presence improved nurses' time management for other tasks, and increased patient cognitive stimulation and safety.<br><br>Continual presence of volunteers during busy times afforded nurses the opportunity to attend to other tasks and overcome the difficulty of competing demands in acute settings.<br><br>Volunteer presence to patients' need for cognitive stimulation prevents further decline and potential behavioural and psychological symptoms.	Patients with dementia and delirium require increased time for explanations and reinforcement of instructions, which volunteer presence ameliorated the constant need. (M, O)<br>Support and comfort provided by volunteers in activities that weren't medically and nursing-oriented increased patient stimulation. (M, O)<br>Nurses had a greater peace of mind knowing that patients were at a lower risk of injury because the volunteers were there supervising them. (M, O)	(Ervin & Moore, 2014)
Improved knowledge reduced stress and perceived workload.<br><br>A bottom-up approach engaging the IPU team helped to define the perceived barriers and challenges for people with dementia and delirium in the IPU setting.	Pre-survey primary themes: Lack of knowledge causing stress and increasing workload, and a difficult physical environment. (C)<br>Post survey: improvement in nursing knowledge and confidence consequently reduced stress and perceived workload. (M, O)<br>The physical environment of the IPU has not been addressed. (C)<br>Establishment of resource roles would be essential to lead the ongoing development of the initiative and enable the continuous monitoring and evaluation of the quality improvement cycle. (M, O) A bottom-up	(Bolton & Loveard, 2016)

Initial CMOC	CMO Code	Evidence studies
Establishment of resource roles would be essential to lead the ongoing development of the initiative and enable the continuous monitoring and evaluation of the quality improvement cycle.	approach engaging the IPU team helped to define the perceived barriers and challenges for people with dementia and delirium in the IPU setting. (M, O)	
Multifaceted educational intervention reduced the early and overall incidence of delirium and resulted in improved objectively measured staff knowledge of delirium and their management of risk factors in patients.<br><br>Reinforcement or using reminders can work at the point of time, but could also warn over time.<br><br>Using delirium champions provided a sense of ownership, motivation, and an initiative role, contributing to the effectiveness of the program.<br><br>Case-based discussion and accessibility of resource staff improved clinical practice.<br><br>Some aspects of practice were not improved by education, particularly in a population with non-English speaking patients, i.e. lack of cognitive testing and overlap with symptoms of dementia or depression. Potential ways to remedy this would be to routinely screen the cognition of all older patients and then check with family or carers; this may require interpreters.<br><br>Clear advantages of this educational intervention include its appeal to staff, local ownership, simplicity, low cost and transferability.<br>The educational sessions were delivered in time already allocated to staff education and are standard duties expected of senior clinicians.<br><br>Regular sessions with delirium resource staff, their accessibility and high visibility were important keys to the success of the intervention.	The improvement in the functional status was a key benefit of the intervention, given the focus on risk factors: mobilisation, adequate pain treatment and removing attachments, which all facilitate function. (M, O)<br>Audit reminder stickers placed in patient files at admission led to a higher rate of initial risk management by staff, which was reflected in a significantly lower incidence of delirium at this time point. (M, O); however, this reinforcing strategy may have waned over time. (M, O)<br>Through weekly meetings with delirium resource staff, posters, and the daily presence of the research team on the ward raised the profile of delirium. (M, O)<br>The presence of delirium champions was likely to have contributed to the program's effectiveness by virtue of their presence giving local ownership to the initiative, and reinforcing, motivating and supportive role. (M, O)<br>Staff feedback highlighted the value of the case-based discussions in improving their clinical practice and the accessibility of resource staff. (M)<br>Despite objective gains in knowledge, staff did not self-rate improvement following the intervention in identifying delirium and distinguishing it from dementia, reviewing medication charts, screening for sensory and cognitive impairment and talking to families and carers about delirium and cognition. (M, O)<br>The lack of staff confidence in discussing cognition and delirium with families and carers represents a missed opportunity to gain information about the patient. (M, O)<br>Both nursing and medical staff identified that they did not routinely assess cognition and optimise patients' medications (two roles often undertaken by medical staff). (M) Staff detection rate of delirium remained poor pre- and post-intervention. (O) This observation suggests that education may be insufficient to address this aspect of practice, particularly in a population with many non-English speaking patients. (C)	(Wand et al., 2014)

Initial CMOC	CMO Code	Evidence studies
The GRAM software provided a mechanism for prospective monitoring of residents during the period in which residents are at the highest risk for ADEs (the first 30 days of nursing home stay).<br><br>The software generated reports for nursing staff's assessment, pharmacists' monitoring and recommendations to reduce ADEs.<br><br>The use of GRAM has the potential to prevent or resolve delirium in residents newly admitted to NH.	Nursing assistants observed and documented on the flow record if symptoms were observed; they notified the nurse. (M)<br>For new admissions, the reports were sent directly to the assessment nurse within 24 hours so that they could identify problems for residents at risk and implement monitoring plans. (M) The consultant pharmacists were on-site once every 30 days to conduct federally mandated drug regimen reviews for every resident. (M)<br>Consultant pharmacists were 4 times as likely to recommend a dose change or discontinue, or monitor a change in residents who triggered the falls or delirium RAP. (O)<br>Although the results did not show a difference in the reduction of mortality and hospitalisation rates, a clear effect on reducing potential delirium onset was observed. (O)<br>Although process measures appear promising in pharmacist interventions in NH, changing patient outcomes has proven elusive. (M, O)	(Lapane et al., 2011)
Using the e-learning tool did not have an impact on patient outcomes.<br><br>Knowledge learned from e-learning modules (educational intervention) with no enabling and reinforcing strategies (i.e. guidelines, pocket cards, reminders or feedback) is less likely to influence behaviour change and positive practice.<br><br>Nurses' attitudes and motivation about the e-learning tool could potentially hinder a successful change in clinical practice.	No significant difference in the overall proportion of delirious patients and in-hospital mortality. (O)<br>No significant differences in the proportions of nurses' ability to correctly identify dementia, hyper/hypoactive delirium and DSD before and after the e-learning intervention. (O); no differences in nurses' delirium knowledge. (O) The reason could be that Geriatric nurses' baseline recognition and knowledge levels about delirium were already high, likely because of their specific experience with delirious patients compared to non-geriatric wards. (C, O)<br>The majority of nurses were only exposed to the 6 compulsory modules. (M) A lack of completion of all modules might in part explain why the e-learning tool failed to affect delirium severity and duration. (M, O)	(Detroyer et al., 2018)
Lack of individual interest, organisational resources and education opportunities serve as barriers in LTC.<br><br>The lower prevalence of delirium may be due to low detection and the unimportance perceived by staff in LTC.<br><br>Passive attitudes in between the roles and misconceptions about the treatment may be due to the lack of education on sufficient delirium information.	Themes: lack of resources – lack of time, education and organisational approach. (C) If it is not compulsory, the guidelines may not be used by those who are not interested. (C)<br>Tendency to follow mindlines rather than guidelines. (M) - The staff know all the residents' conditions, and every day is the same for them. (C). Staff stated they have seen little delirium here for many years. (C)<br>Passive attitude – delirium tx is the doctor's responsibility, and non-pharmacological interventions are some of the things we can do. (M)<br>Even if delirium is observed, we just notify the doctor about some drugs or refer them to the hospital. (M)<br>Misunderstanding about delirium care in LTC – caregivers don't want to actively find the cause or treat it. (C) Delirium care is always aggressive treatment. (C)	(Jeong et al., 2020)
After efforts in engaging multidisciplinary clinicians, addressing barriers and enablers, and supporting user involvement, the outcomes remain limited and mixed.<br><br>Using an audit as a means of supporting change and reminding clinicians that they are being held accountable.	Significant decrease in delirium rate, and compliance with the use of the screening tool. No difference in the length of hospital stay and duration of delirium. (O)<br>Efforts were made to engage multidisciplinary clinicians and address barriers and enablers during the development of the intervention. (M)<br>Despite the effort at all levels, the project showed mixed results. (O)<br>A senior nurse was allocated to the use of the delirium screening tool as a portfolio project, which led to regular auditing and feedback in clinical handover meetings. (M, O)	(Oberai et al., 2021)

Initial CMOC	CMO Code	Evidence studies
Engagement from the senior management level has the potential to create significant improvements.		
Recreational activities targeting cognitive domains provided significant positive effects on delirium severity and attention and insignificant fewer days of delirium.	Participants' engagement and satisfaction data showed satisfaction and recommended it to other facilities. (O)<br>Differences in delirium severity and attention were significant(O).<br>Participants were able to engage in the prescribed recreational activities for 30 days, and there didn't appear to be any systematic preference for one kind of activity over the others. (M, O) All staff believed the activities fit well into their daily routine. (C, M)	(Kolanowski et al., 2011)
The incidence of delirium can be prevented in a frail hip fracture population by multidisciplinary IGCT.<br><br>The effect of IGCT on the duration and severity of delirium was no different.	No significant differences in care given to the groups, except that intervention participants received more occupational therapy and opioid pain medication than controls. (M)<br>Incidence of postoperative delirium was 30% lower in intervention participants. (O)<br>No differences were seen in the duration and severity of postoperative delirium. (O)<br>More control group had cognitive decline than intervention group. (O)	(Deschodt et al., 2012)
Solicited feedback during education sessions, while implementing and after increased staff adherence to the intervention.<br><br>Mixed staff perceptions about not medicating patients and having strategies to support nurses in this regard are useful, leading to positive feedback and adherence.<br><br>Computerised format screens prompted nurses to adhere to the care standards; decision support tools enhanced diagnostic agreements.	Nurse adherence to assessment screens was 100% and 75% on the sleep protocol screen. (O)<br>Solicited feedback from staff regarding modules during and after the educational sessions, and while they are actually using the screens to increase adherence and enhance workload fit. (M, O)<br>Mixed feedback about not medicating patients and having access to strategies such as sleep protocol for nursing management of delirium, one said the hardest part would be not medicating patients, and another said it would be nice to have strategies besides calling psychiatry to medicate patients. (M, O)<br>Overall, nurses did not have problems using assessment and management screens, and it prompted them to assess and manage delirium using current standards of care. (M, O)<br>Most patients' mean admission MMSE was either improved or no change. (O)<br>Patients and family survey (n=13): 6 family members thought the patient strongly benefited from the intervention, 2 thought not, and 5 were uncertain. (O)	(Fick et al., 2011)
Individualised cognitive stimulating activities, delivered with high fidelity combining physical and occupational therapy in rehabilitation, did not decrease delirium duration or severity, with a small effect on executive function, favouring intervention and fewer days in PAC.<br><br>Executive function may be an important cognitive domain that reflects central nervous system integrity after delirium and a sensitive indicator of the effectiveness of delirium interventions.<br><br>Although the intervention did not reduce delirium, it may have a role in future delirium prevention	No significant difference between groups in - Time to first remission of delirium, 6.88 days (Intervention) and 7.39 (Control), symptoms of delirium on discharge or completion of intervention period, and severity of delirium. (O)<br>Significant difference in executive function and constructional praxis, length of stay favoured the intervention. (O)<br>Discharge location: more intervention participants returned to the community, and fewer were institutionalised than in the control group. (O)<br>Most participants had no or partial delirium recovery 3 months after enrolment, despite significant improvements in global cognition and function. (O)	(Kolanowski et al., 2016)

Initial CMOC	CMO Code	Evidence studies
by strengthening important cognitive domains in executive function and providing cognitive reserve to protect against delirium risk factors.		
Nurses developed CogChamp, which resulted in increased cognitive assessment, analgesia, change in benzodiazepine use and the development of resources for patients with CI.<br><br>However, the data do not indicate whether analgesia use was optimal.<br><br>Organisational factors associated with efficiency and risk have been linked to how nurses ration their care tasks so that only essential or emergency care tasks are prioritised, and other discretionary tasks, while significant to the health and well-being of confused patients, are often never completed.<br><br>Non-completion of care tasks was due to no staff member assuming responsibility for the tasks, and also due to a funding issue and a lack of priority effort by leadership.<br>Nurses' secondment of CogChamps to work as facilitators effectively overcame some of the time constraints and enabled the progression of action plans.<br><br>The support from NUM and the research team, and the development of ward-specific action plans led by CogChamps ensured each plan was tailored to address each ward's specific requirements and circumstances.<br><br>Development of the plans is likely to have increased the CogChamps' awareness of the need for change, which is important in motivating implementation efforts.<br><br>The project's impact waned following completion and is likely to continue to decline without	A significant increase in cognitive assessment using a tool and an informal assessment was documented, such as 'patient confused'. (O)<br>A significant increase in analgesia and a change in benzodiazepine administration, but declining over time, follow-up. (O)<br>No change in the number of patients who received antipsychotic medication. (O)<br>While all wards adopted resources to improve care, the use of resources or tools by individual nurses was discretionary. (M)<br>Some practices did not change significantly, i.e., meal-time difficulty, nurses introducing themselves, and addressing patients' names. (O)	(Travers et al., 2018)

Initial CMOC	CMO Code	Evidence studies
continued emphasis on delirium as an important issue.		
A significant reduction in the use of benzodiazepines as a sleeping pill, as well as rescue medication, may have contributed to an improvement in the severity of delirium.<br><br>Clinical leadership is important to staff adherence for practice development.<br><br>The time frame of 5 days to conduct the delirium screening by ward nurses was revealed to be too short, as only 81.6% of delirium cases were detected. However, nurses agreed as they wanted to avoid unnecessary paperwork. This could result in a delirium detection rate.	Delirium incidence was lower but not significantly so in the intervention group. (O)<br>Positive effect of the severity of delirium on adherence to wards. (O)<br>No significant change in the duration of delirium (mean 3-4 days). (O)<br>A shift from haloperidol to atypical neuroleptic quetiapine in the intervention group. (O)<br>A significant decrease in lorazepam use in the intervention group. (O)<br>Non-adherence rate of 57.1% on one ward was caused by a head nurse who restarted the discussion and criticised the algorithm of assessments in front of his team, resulting in an overall non-adherence to the protocol. (M, O)<br>Nurses had the lead in delirium management. (M)	(Hasemann et al., 2016)
Focused assessment, exemplified by the themes of knowing the patient's baseline, knowing the patient's interests and values, may help to alleviate the associated issues of caring for individuals with dementia and delirium.<br><br>By implementing focused assessment of patients' baseline, interests, and values, we can allow older adults with dementia and delirium to communicate and participate to the fullest extent possible as members of the healthcare team.<br><br>Tailoring intervention incorporates the themes of enhancing sensory abilities to communicate, individualising cognitive stimulation, and enhancing behavioural approaches to comfort and sleep. Utilising person-centred care approaches may help to ease the burden when staff are trained in these methods, and the environment is modified for the inclusion of PCC principles.<br><br>Small changes in the provision of low-cost hearing amplification devices or whiteboard communication templates can be readily used for older adults to facilitate participation to the	Five themes:<br>Knowing the patient's baseline -the ability to assess for delirium rests upon understanding prior cognitive and physical function. (M, O) Since establishing a baseline in an acute care setting is often difficult, there is a need to talk with family members and/or the facility to determine the patient's normal function and cognition. (M)<br>Knowing the patient's interests and values – this helps a patient hold on to his sense of self. (M, O) It also provides a means of establishing a connection and bond of trust between nursing staff and patients, allowing for individualised care. (M, O)<br>Enhancing sensory abilities to communicate – the use of an amplified hearing device in conversations improves the ability of patients to communicate and allows them more understanding and input into their medical care. (M, O)<br>Individualised cognitive stimulation – tailoring activities to the patient's specific interests and cognitive stimulation 'activity kits', patients were more likely to remain engaged and show cognitive improvement. (M, O)<br>Behavioural and non-drug approaches to comfort and sleep – nurse facilitators advocated review of the medication list of each patient and made as-needed suggestions for changes to the unit champions and nursing staff, limiting medications that may have harmful effects in patients with dementia. (M), The intervention encouraged assessing for and meeting unmet needs with non-drug approaches prior to administering drugs. (M)<br>Barriers to person-centred care in this context – failure to recognise the patient first (nurses overlooked the patient's opinions and ability to make their own decisions -approached the family to find out what the patient liked when preference could have been asked of the patient first. (M, O)<br>Not enough time (nurses and unit champions reported being busy, having no time to implement non-drug alternatives, cognitive testing, or participate in the rounds). (M, O)	(Yevchak et al., 2017)

Initial CMOC	CMO Code	Evidence studies
fullest extent possible in healthcare decisions and activities.<br><br>Barriers to the implementation of tailored interventions were seen in the theme of lack of time. Small person-centred changes can be incorporated into routine clinical care and begin the movement of an institution's culture toward PCC.		
Barriers to implementing the VDDC program – perception that caring for patients with dementia/delirium is difficult, threat to volunteer safety, need for clearly defined volunteer roles and responsibilities and volunteer attrition and availability.<br><br>The clinical staff did not have time or capacity to provide the level of psychosocial support required in the program, and volunteers addressed an unmet care need for the patients.<br><br>Enablers included the need to utilise the skills and knowledge of volunteers, the opportunity to make the program available to all patients, the importance of patients with no family or friend support, the opportunity for volunteers to focus on the medical aspects of care and recognition from staff that volunteers will improve the care experience for patients.	Capability: The majority of volunteers understood that the behaviour was a consequence of cognitive issues and was not a personal attack. (M)<br>One doctor and the registrar highlighted the need to ensure the emotional safety of volunteers; they need to be trained. (M) There are underlying racist or gender things that come up, and to be able to deal with them and know how to respond to those things. (C, M)<br>The NUM spoke about ensuring the volunteers understood their scope of practice and the need to ensure that they did not utilise any medical skills or knowledge they had from their study or professional roles(M).<br>Opportunity: The NUM cautioned that having non-qualified volunteers provide care may cause some issues with the unions and recommended keeping the program simple. (C)<br>Volunteers can focus on the non-medical aspect of care, such as social and emotional support. (M) Particularly important for patients with no family or friend support. (M)<br>Volunteers recognised the need to debrief about their experiences and to flag any patient issues with a staff member. (M)<br>Motivation: recognition from staff that volunteers will improve the care experience for patients. (M, O)<br>Attrition has been a problem, getting them engaged based on prior experience, particularly in relation to training volunteers and then having them not be available or leave after a short duration. (M)	(Ayton et al., 2020)
The process of guideline adaptation can lead to engagement and capacity building, with a participatory approach, developing a community of practice.<br><br>Non-pharmacological management can be implemented once awareness is raised through information modules/resources.<br><br>Presenting pharmacological interventions as a framework rather than a prescriptive guideline on prescribing that removes physician autonomy makes it possible to nudge practice change.	The guideline implementation took 12 months (23 plus 7 face-to-face sessions, 3 online e-learning). (C, M). The survey showed that all participants either strongly agreed or agreed that the training was sufficient for them to follow the guidelines in daily practice. (O) Four occurrences of nursing documentation of first-line use of non-pharmacological interventions for relief of delirium symptoms or related distress, and 60% less scheduled antipsychotic use. (M, O)<br>Participants' perceptions of guideline implementation reflected the temporal nature of changing practice. (M, O) changing practice involved incorporating prior knowledge or experience, confronting challenges during the change and sustaining change. (M) Some participants' prior knowledge and extensive experiences of delirium informed their current practices, contributing to viewing the guideline as either basic or supplemental to past experiences. (M, O)<br>Limited staff presence at night was identified as a challenge. (M)<br>Participants noted elements of their future practice improving or, conversely, not anticipating changes but instead reinforcing their current practices. (M, O) Making changes to current practice was perceived to be a collaborative effort, enabling contributions from unit staff,	(Bush et al., 2022)

Initial CMOC	CMO Code	Evidence studies
Multimodal education interventions with narrative rather than solely relying on printed or electronic educational materials for guideline dissemination led to the implementation of a novel modular guideline for the entire interprofessional team.<br><br>Incorporating participants' prior knowledge and experience contributed to and reinforced current practice.<br><br>A successful guideline implementation requires behaviour change from more than a single professional group – a collaborative effort.<br><br>Contextual factors of stability within the project team, advanced nursing practice leadership, protected time and financial support for nurse attendance at educational sessions contributed to successful guideline implementation.<br><br>Information that was developed by the team encouraged a sense of ownership, contributing to engagement.	volunteers, and family caregivers to effectively recognise and manage delirium. (M)<br>The family information booklet engaged the team, encouraged thinking about delirium and gave the team ownership of the resource as it incorporated their feedback. (M, O)<br>The face-to-face small group sessions enabled presenters to gauge the current knowledge of the attending participants. (M, O)	
Even in the specialist group, considerable variation in perception of delirium and confidence in diagnosis and management according to different clinical presentations of delirium.<br><br>Confidence and diagnosis of delirium with BPSD are considered especially challenging.<br><br>The pre-workshop highlighted the lack of precision in psychiatrists' awareness of diagnostic criteria for delirium.<br><br>Baseline differences in the willingness to use antipsychotics may reflect the differing subspecialty backgrounds of respondents and also likely relate to varying appreciation of research that addresses risks and benefits of pharmacotherapy in delirium.	Pre-workshop(O)<br>90% of respondents perceived delirium as a condition with high relevance to psychiatry. The level of confidence in the diagnosis was modest (82% >5,37%>8). Confidence in distinguishing delirium from dementia (median7), depression (median7), behavioural and psychological symptoms of dementia (median6), and psychosis (median7). Only 9 (15%) participants were able to correctly identify DSM-5 delirium criteria without omissions or commissions. The correlation between accuracy rating and perceived self-confidence in diagnosis was only modest. The level of confidence in managing different clinical presentations was higher for patients with hyperactive delirium than hypoactive delirium. For hyperactive presentation, 22% indicated they would avoid pharmacological therapy. 94% indicated they would typically use an antipsychotic agent as first line, three indicated a preference for benzodiazepine treatment monotherapy, and five indicated combined antipsychotic-benzodiazepine therapy. In respect of antipsychotic agents, 38 indicated a preference for haloperidol (79%), quetiapine (n=13%), and risperidone (n=8%). For hypoactive presentation, 72% indicated they would avoid using pharmacotherapy. A preference for using pharmacological strategies was more frequent for hyperactive than hypoactive presentation ($p<0.001$).<br>Post-workshop. (O)<br>After the workshop, the perceived relevance of delirium to psychiatry increased. Significant improvement in the perceived confidence in delirium diagnosis, along with the range of key differential diagnoses	(O'Regan et al., 2019)

Initial CMOC	CMO Code	Evidence studies
The outcomes pertained only to short-term improvements in attitudes, perceptions and knowledge, and it is unclear whether the workshop will have any enduring effect on clinician behaviour or patient outcomes.	(p<0.001). The accuracy index in terms of DSM-5 diagnostic criteria significantly improved. The appreciation of inattention as a mandatory feature increased to 100% while the endorsement of disorientation and psychomotor disturbance as mandatory features reduced. The perceived confidence in treating delirium improved for both hyperactive and hypoactive presentations (p<0.001). For hyperactivity, 8 indicated a preference for non-drug treatment, and 5 indicated a preference for antipsychotic use. For the hypoactive case, 30 indicated a preference for no drug treatment pre-workshop, and 13 switched to indicating their preference to include antipsychotic treatment. (O)	
A highly engaged nursing unit manager and clinical nurse facilitator contributed to the coordination of the program.<br><br>Knowledge gained from different ways of engagement through online learning, discussion and simulation attributed to nurses' incorporation of knowledge from these experiences into their personal construction of knowledge.<br><br>The improvement of knowledge is attributed to the combination of knowing and meaning approaches through online modules and discussion groups.<br><br>The sustained level of knowledge over time following the completion of the program suggests that the nurses may have developed a shared understanding of delirium prevention and care, with potential for social transformation.	While the three-element approach was relatively straightforward to introduce and sustain over time, coordination work was required. (C) The coordination relied on a highly engaged nursing unit manager and an experienced clinical nurse facilitator. (M) 35 nurses completed the online module(83%), 36 attended at least one discussion sessions(85%), and 20 completed one simulation session(48%). (O) There was an overall significant improvement in knowledge (p=0.001), specifically T0-T2(p=0.03) and T0-T3(p=0.003). 92.6-100% of participants responded correctly to some questions and maintained over time. (O) However, some questions remained sub-optimum. (O) Question relating to comorbidities and the senses remained low, and a family history of dementia was particularly difficult, with only 50% of respondents providing the correct response. (O) The improvement in correct scores on the knowledge survey over time suggests that the knowledge gained through engagement in online learning, discussion, and high-fidelity simulation may be attributed to the different ways that nurses incorporated knowledge from these experiences into their personal construction of knowledge. (M, O) Relations between nurses who were engaging in educational activities (newcomers) and other nurses(old timers) were changing, with the potential of changing practice. (M, O) Nurses appeared to have strong taken-for-granted beliefs about risk factors for delirium that were not changed by these learning experiences. (O)	(Grealish et al., 2019)
Improved previous lack of knowledge in risk subscale: recognition, predisposing factors and medication that can precipitate delirium.<br><br>No difference in knowledge of delirium, as it was already high pre-education.	Audit: The use of a cognitive assessment tool increased from 8.5% to 43%. (O)<br>Questionnaire: Significant increase in awareness of the appropriate use of the Montreal Cognitive Assessment for dementia(p=0.015) for dementia. Significant increase in the combined knowledge and risk subscales' total score. Nurses demonstrated a significant increase in delirium knowledge, with a pre-intervention mean score of 60.8% and a post-score of 65.4%(p=0.045). (O) Participants' knowledge of delirium pre- and post-education intervention did not reach statistical significance, with the level of knowledge already high pre-education. (O)	(Ewens et al., 2021)
Approaches of 1. Institute mandatory delirium education training focused on screening at nursing admission and shift assessment. 2. Implement the EMR delirium order set in which the bedside nurses can activate nonpharmacological orders,3. Establish an interprofessional delirium consult service,	Clinical support (M): delirium consult team (psychiatrist, geriatrician, nurse). Computerised patient record system(triggered by nurse assessment, free-standing order). Evidence-based CPRS response order set(per policy, RN can activate non-pharmacological interventions). Nursing delirium screening scale (embedded in nursing admission and shift assessment). Unit-based delirium nurse champion. Notification of PCP at time of discharge. Following the completion of the virtual dementia tour(VDT). (M) Participants experienced significant changes in their sensitivity to the impact of cognitive impairment on patients' ability to function in everyday life. (O) Participants who completed the 4-month	(Solberg et al., 2021)

Initial CMOC	CMO Code	Evidence studies
contributing to the positive outcome of the education program.<br><br>The program showed significant changes in nurses' attitudes about caring for patients with delirium, including increased confidence in recognising and treating delirium.	post-program survey(n=43) reported increased self-confidence and ability to recognise signs and symptoms of delirium, administer a delirium screen, implement nonpharmacological delirium intervention and provide overall better care. (O)	
The paucity of knowledge and understanding of delirium prevention, particularly among junior doctors, nursing and care staff, posed challenges for implementation.<br><br>The term delirium was generally not used; instead, the word confusion was used among care staff.<br><br>Low coherence of delirium awareness across the clinical sites, delirium prevention was not perceived as meaningful.<br><br>In the hectic nature of the acute environment, care priority was given to what was immediately presented.<br><br>Nutrition, fluids, sensory aids and mobility depend on the engagement of nurses and care staff.<br><br>The local policies and priorities contributed to the existence of a care ethos and designated activities to stimulate cognition during hospital stays.<br><br>The training and involvement of volunteers in the care was not a static document that would be subject to regular review and change based on action and outcomes.	Knowledge and awareness of delirium:<br>Although junior doctors might be familiar with the term 'delirium', knowledge-based understanding was seen to have improved among registrars specialising in care of older people. (M, O), There was less confidence that such knowledge was routinely translated into action to prevent delirium or manage it when it occurred. (C, O)<br>For nursing and therapy staff, delirium had not featured in their professional training. (C) Among all staff, delirium was not included as part of mandatory training or in-service education programmes. (C) Low salience attached to delirium and delirium prevention in policy and practice. (C)<br>Nursing, therapy and care staff generally did not use the term delirium; instead, confusion or acute confusion was more typically employed in elderly care wards. (C) - It's just that perhaps they don't recognise it as delirium. They don't put a label on it (Doctor said) (C)<br>The conflation of delirium and dementia by staff was a source of heightened anxiety and perplexity among caregivers/relatives, as the sudden change of behaviour was not understood by staff. (C, O)<br>Aggression or refusal to participate in treatment could be interpreted as a lack of engagement, resulting in the patient being perceived as unsuitable for rehabilitation or berated by staff for inappropriate behaviour. (C, O)<br>Lack of understanding about delirium and investment at an organisational level with respect to education and training, in NPT terms, had low coherence. (C)<br>Delirium prevention:<br>Given the low coherence of delirium among staff groups across sites, delirium prevention was not perceived as meaningful. C) Even senior staff had initiated action to increase awareness, but this didn't inform assessment and care practices; it was not in the foreground of people's minds (geriatrician said).<br>Current ward routines and practices:<br>The hectic nature of ward life had the consequence that routine practice was described by staff as being primarily directed at responding to what was immediately presented, with priority given to diagnostic, observational and interdisciplinary assessment and care planning. (C)<br>Nutrition, fluids and sensory aids:<br>Although nutrition and fluid intake were viewed as basic care by ward staff, they were primarily delivered by healthcare assistants. (C) The importance that senior nursing staff attached to care tasks affected both the value attributed to them by junior staff and the extent to which they pitched in to provide assistance. (C, M)<br>Mobilisation:<br>The engagement of nurses and healthcare assistants routinely in mobilisation work in either enhancing or supportive roles was viewed by staff as essential to sustaining mobility among patients. (M, O)<br>Orientation and communication:<br>Sustained or prolonged engagement of patients by staff was absent in all sites. Development teams remarked that this was neither feasible nor valued in the context of the priority attached to moving patients quickly through the system. (C, M)	(Godfrey et al., 2013; Young et al., 2021) project 1

Initial CMOC	CMO Code	Evidence studies
	Practices vary depending on local policies and priorities, the physical environment in which care was delivered and the existence of a care ethos that placed high value on social engagement and care or cognitive stimulation. (C, M)<br>Volunteers:<br>Sustaining volunteer involvement depended on the commitment, tenacity, skills and abilities of individual volunteers and mutual support provided to each other through informal networks. (C, M)	
Critical to implementation was the combined and coordinated involvement of the triumvirate of a named, individual driver at a senior level whose professional authority and vertical networks legitimised the work of POD implementation in the face of competing priorities.<br><br>A ward-based facilitator is typically the ward manager who provides support and encouragement to legitimate staff time devoted to POD and extends its reach to the wider staff team.<br><br>Partial implementation and failure to engage with the PODv1 in some wards posted issues in addition to leadership.<br><br>The findings informed four criteria that are going to be able to implementation of the complex intervention:<br>1. Commitment of the senior nurse, ward manager and volunteer service manager.<br>2. A named person to drive implementation forward.<br>3. Dedicated time of a senior experienced nurse to lead the implementation.<br>4. Adequate staffing levels.<br><br>An early phase of delivery is critical to continuance.<br><br>For volunteers, factors that appeared to be important are:<br>1. Ongoing recruitment of volunteers with an expressed interest in the POD programme.<br>2. A comprehensive POD-specific training.<br>3. A robust support system.	Implementation planning:<br>Critical to the engagement of ward staff and volunteers in the implementation was involvement and direction provided by those with authority, legitimacy and resources to make change happen. (M) Features of the change management process to engage implementation team members in seeing the need for change were initially a concern because of the time required, but later became valued as a lens to see current practice and identify taken-for-granted practices that required attention and reinforce what was positive that could be built. (M, O)<br>Each ward conducted the audits and developed its own documentation for identifying delirium risk, delirium care plans, job descriptions for volunteers and systems for communication between staff and volunteers. (M)<br>Creative approaches to delivering training to ensure inclusion of all staff, i.e., multiple short sessions during early morning break and reinforcement through discussion at handovers. (M)<br>The flexible approach was based on recognition that my ward is different from your ward. (C) Fostered creativity and problem-solving approach and active decision-making by staff in how to make change happen, contributing to staff ownership of change. (M)<br>Delivery phase:<br>Several sites developed new ways of responding to needs around communication and stimulation, i.e., reminiscence work, playing games, music, and books. (M)<br>Education sessions, observation and structured review of current practice provided helpful and facilitated empathic connection with the experience of patients with delirium as opposed to just seeing a problem patient. (M, O)<br>Slowly noticed at handover that staff wouldn't say Mrs X was confused, but talked about whether the patient may have a delirium. (O) They also reported that nursing and care staff were more patient and would spend time talking to the patient behaving badly and seeking to ease their confusion. (M, O)<br>Volunteers:<br>A limited number of volunteers was available. Despite attention to introducing volunteers to ward staff prior to delivery and POD training, they were initially anxious and unclear about their role and lacked confidence about how to approach patients. (M, O)<br>About half the volunteers initially recruited did not sustain their involvement over the 6 months of PODv1 delivery. (O)<br>It was considered inadequate support at the ward level to be a key factor in volunteer attrition, particularly for less confident and inexperienced volunteers. (M, O)	(Young et al., 2021)<br>Project 2

Initial CMOC	CMO Code	Evidence studies
The recruitment rate was lower than anticipated. The major barrier to recruitment was the inability to conduct a baseline CAM assessment to exclude prevalent delirium.<br><br>The rate of delirium incidents was lower than anticipated. This suggested some variation in either individual RA delirium assessment performance or differences in local care environments that influenced the development of delirium.<br><br>The two groups had a similar incidence of delirium, severity, duration, first episode of delirium, length of stay, and fall.<br><br>Used a range of methods, including non-participant observations of care delivery, case note reviews and examination of staff-completed delirium assessment and care plans to obtain as complete a picture of intervention fidelity as possible.	Completion rate of CAM assessment was 89.7% during the first 10 days of recruitment and 81.6% at 30 days. (O) The recruitment was achieved in 8 hospitals/16 wards over 6 months; nearly ¾ of patients in the elderly care and orthopaedic trauma wards were at risk. Of the 8 wards, 2 achieved >80% compliance rate, 5 achieved 51-79%, and 1 rated as low <50% compliance rate. (O)<br>8% of the 713 participants developed new-onset delirium within 10 days of recruitment. Although the POD arm had lower odds of developing delirium, there was no significant difference. (O)<br>The POD group had a higher average resource use for every health care resource except GP, psychiatrist, psychologist or counsellor visits, resulting in high costs(O). However, observed in lower delirium incidence and rate reduction, the POD intervention was cost-effective. (O)<br>Severity, duration and time to first episode of delirium were similar between the two groups. (O)<br>Length of hospital stay (9.7/9.8 days) and fall (19/20) between groups were similar(O). Deaths between sites from 5-22. (O) A larger proportion of patients in POD (71%) were discharged home than in the control (67.4%). (O)<br>Patient-reported outcomes (clinical anxiety scale, Geriatric depression scale) showed little difference. (O)	(Young et al., 2021)<br>Project 3
